# Role of Recurrent Hypoxia-Ischemia in Preterm White Matter Injury Severity

**DOI:** 10.1371/journal.pone.0112800

**Published:** 2014-11-12

**Authors:** Matthew W. Hagen, Art Riddle, Evelyn McClendon, Xi Gong, Daniel Shaver, Taasin Srivastava, Justin M. Dean, Ji-Zhong Bai, Tania M. Fowke, Alistair J. Gunn, Daniel F. Jones, Larry S. Sherman, Marjorie R. Grafe, A. Roger Hohimer, Stephen A. Back

**Affiliations:** 1 Department of Pediatrics, Oregon Health & Science University, Portland, Oregon, United States of America; 2 Department of Physiology, Faculty of Medical and Health Sciences, University of Auckland, Auckland, New Zealand; 3 New Zealand Genomics Ltd./Bioinformatics Institute, School of Biological Sciences, University of Auckland, Auckland, New Zealand; 4 Division of Neuroscience, Oregon National Primate Research Center, Beaverton, Oregon, United States of America; 5 Department of Cell and Developmental Biology, Oregon Health & Science University, Portland, Oregon, United States of America; 6 Department of Pathology, Oregon Health & Science University, Portland, Oregon, United States of America; 7 Department of Obstetrics and Gynecology, Oregon Health & Science University, Portland, Oregon, United States of America; 8 Department of Neurology, Oregon Health & Science University, Portland, Oregon, United States of America; Robert Debre Hospital, France

## Abstract

**Objective:**

Although the spectrum of white matter injury (WMI) in preterm infants is shifting from cystic necrotic lesions to milder forms, the factors that contribute to this changing spectrum are unclear. We hypothesized that recurrent hypoxia-ischemia (rHI) will exacerbate the spectrum of WMI defined by markers of inflammation and molecules related to the extracellular matrix (hyaluronan (HA) and the PH20 hyaluronidase) that regulate maturation of the oligodendrocyte (OL) lineage after WMI.

**Methods:**

We employed a preterm fetal sheep model of *in utero* moderate hypoxemia and global severe but not complete cerebral ischemia that reproduces the spectrum of human WMI. The response to rHI was compared against corresponding early or later single episodes of HI. An ordinal rating scale of WMI was compared against an unbiased quantitative image analysis protocol that provided continuous histo-pathological outcome measures for astrogliosis and microglial activation. Late oligodendrocyte progenitors (preOLs) were quantified by stereology. Analysis of hyaluronan and the hyaluronidase PH20 defined the progressive response of the extracellular matrix to WMI.

**Results:**

rHI resulted in a more severe spectrum of WMI with a greater burden of necrosis, but an expanded population of preOLs that displayed reduced susceptibility to cell death. WMI from single episodes of HI or rHI was accompanied by elevated HA levels and increased labeling for PH20. Expression of PH20 in fetal ovine WMI was confirmed by RT-PCR and RNA-sequencing.

**Conclusions:**

rHI is associated with an increased risk for more severe WMI with necrosis, but reduced risk for preOL degeneration compared to single episodes of HI. Expansion of the preOL pool may be linked to elevated hyaluronan and PH20.

## Introduction

Critically ill prematurely born infants are particularly susceptible to hypoxic-ischemic cerebral white matter injury (WMI). WMI is the leading cause of cerebral palsy (CP) in survivors of premature birth and contributes to a wide range of life-long neurobehavioral disabilities [1]. This developmental predilection for WMI is related to vascular maturational factors that include disturbances in cerebral auto-regulation [2], as well as an enriched population of late oligodendrocyte progenitors (preOLs) that populate the white matter during a broad high-risk period for injury [3]. Hypoxia-ischemia (HI) results in graded WMI that becomes progressively more severe with a more prolonged duration of HI [4]. Quantitative, anatomically-defined cerebral blood flow studies demonstrated that the spatial topography of ischemia is not sufficient to define the distribution of selective WMI [5], which typically displays a low burden of necrosis in human [6] and fetal sheep [7]. Rather, the topography of this diffuse WMI is defined by the density and distribution of susceptible preOLs within the ischemic territory [4].

Although preterm infants are commonly at increased risk for recurrent hypoxia-ischemia (rHI) during intensive care, the contribution of rHI to the progression of WMI and the burden of necrosis has received limited study. Large cystic necrotic lesions were the major form of WMI in prior decades [8]. However, advances in neonatal care have coincided with a pronounced shift to milder forms of WMI characterized by occult microscopic necrotic lesions [6] that are typically not detected, but are resolved by high field MRI [7]. White matter necrosis contributes to CP, because of degeneration of preOLs and axons, which are required for normal myelination [9], [10]. Necrotic WMI appears to contribute to neurobehavioral disabilities via retrograde axonal degeneration that causes secondary neuronal loss in multiple gray matter structures [11]–[13]. In contemporary human cohorts, diffuse WMI more frequently displays mild axonopathy and an expanded, chronically dysmature pool of preOLs in and near astrogliotic lesions [6].

Studies in rodents, sheep, and humans have demonstrated a central role for disturbances in preOL maturation in the pathogenesis of myelination failure [3]. After HI, preOLs degenerate in the preterm equivalent neonatal rat in two temporally distinct waves. The first is caspase-independent and the second is caspase-driven [14]. During the initial phase of WMI, preOLs proliferate [15], but subsequently fail to differentiate to myelin-producing cells [6], [7], [14]. Multiple molecules appear to act in concert in chronic white matter lesions to prevent preOL maturation and normal myelination [13]. Among these is hyaluronan (HA), a glycosaminoglycan that derives from reactive astrocytes and accumulates in the extracellular matrix in human preterm WMI [6] and in adult demyelinating disease [16], [17]. PreOL maturation is blocked in vitro and in vivo by high molecular weight forms of HA, which are digested to bioactive molecules by a membrane-associated hyaluronidase, PH20 that displays enhanced expression in adult demyelination [18]. Pharmacological inhibition of hyaluronidase activity promotes OL maturation in vitro [17], [18] and myelination in vivo, which is accompanied by enhanced nerve conduction [18].

While multiple studies have defined the pronounced susceptibility of preOLs to WMI [3], the susceptibility of dysmature preOLs to recurrent HI has received little study. To define the effect of rHI on preterm fetal WMI severity, we employed a preclinical model of WMI in the instrumented fetal sheep that closely replicates major features of human WMI [20]. In this model, ischemia is combined with maternal hypoxemia, and a single HI insult generates WMI with a low burden of microscopic necrosis [10], [19]. We adapted this model to generate recurrent HI. The timing of the second HI insult was chosen to coincide with a period at one week after HI when preOLs have widely repopulated the white matter. Prior studies in preterm-equivalent neonatal rats demonstrated that rHI markedly enhances preOL degeneration [14]. We report that rHI is associated with more pronounced necrotic WMI, but an expanded population of preOLs that display reduced susceptibility to cell death.

## Materials and Methods

### Ethics Statement

All survival studies were performed in a core facility of the Oregon Health & Science University (OHSU) Department of Comparative Medicine and adhered strictly to protocols that were approved by the OHSU Institutional Animal Care and Use Committee. Animals were housed in individual stalls, and maintained in a climate-controlled environment on a 12-hour light/dark cycle where they had access to food and water. Ewes were monitored on a daily basis. If ewes showed signs of pain, which could not be managed through normal postoperative care (below), they were euthanized. Ewes were euthanized by an intravenous bolus of a commercially available euthanasia solution (SomnaSol; Butler OH, 10,000 U IV), as recommended by the Panel on Euthanasia of the American Veterinary Association. Separate survival studies were performed at the University of Auckland, Auckland, NZ and were approved by the Animal Ethics Committee of The University of Auckland, [21].

### Animal surgery

Sterile surgery was performed at OHSU on time-bred, twin or triplet pregnant sheep of mixed western breed between 89–93 days of gestation (dGa; term: 145 days). The ewe was initially anesthetized with intravenous ketamine (5 mg/kg) and diazepam (0.13 mg/kg), an endotracheal tube was placed, and anesthesia maintained with 1–2% isoflurane in oxygen (O_2_) and 1–2% nitrous oxide. Maternal end tidal partial pressure of carbon dioxide (PCO_2_) and oxygen saturation (SO_2_) were monitored continuously. A midline laparotomy and a hysterotomy were performed in a sterile field and the fetus exposed. A vinyl catheter was non-occlusively placed in the fetal carotid artery, and an inflatable silastic occluder (4 mm; In Vivo Metrics, Healdsburg, CA) was placed on the brachiocephalic artery [10], [19]. In sheep, the brachiocephalic artery supplies blood bilaterally to the brachial and carotid arteries. To confine the cerebral blood supply to the carotid arteries, the occipital vertebral anastomoses were ligated bilaterally. These anastomoses connect the vertebral arteries, supplied by the thoracic aorta, with the external carotid arteries that are fed by the brachiocephalic artery [22]. An additional vinyl catheter was sewn to the fetal skin to allow for monitoring of the amniotic fluid pressure. After instrumentation, the fetus was returned to the uterus and the uterus was closed by invaginating the uterine wall with a purse-string suture around the catheter ends. One million units of penicillin G were then delivered into the amniotic fluid via the amniotic catheter, and the non-occlusive carotid artery catheter was filled with a 50% solution of heparin in saline. All catheters were exteriorized through a subcutaneous tunnel to the flank of the ewe, and the abdominal wall of the ewe was closed in layers. One-week survival twin surgeries were performed in two groups: in the first group (early HI; n = 6 ewes), one fetus was instrumented and the twin served as the un-instrumented control. In the second group (n = 6), both fetuses (late HI, rHI) were instrumented. In all 2-week and 4-week survival twin surgeries, one fetus was instrumented while the twin served as a control. In 24-hour survival triplet surgeries, one fetus was instrumented while the other two were controls. During postoperative care, ewes were monitored for pain, and were given four doses of the analgesic buprenorphine (0.3–0.6 mg) subcutaneously over the first two postoperative days.

For PH20 expression studies at the University of Auckland, the fetuses of time-mated Romney/Suffolk sheep were instrumented using sterile technique at 93–94 days gestation, essentially as previously described [21]. One fetus was instrumented while the twin served as an un-instrumented control. Animals from successful HI studies at 98–99 days gestation (control n = 4, HI n = 6) survived for 24 h and frontal white matter was snap frozen for RNA expression and RNA-seq studies, described below.

### Cerebral Hypoxia-Ischemia Studies

**Figure 1:**
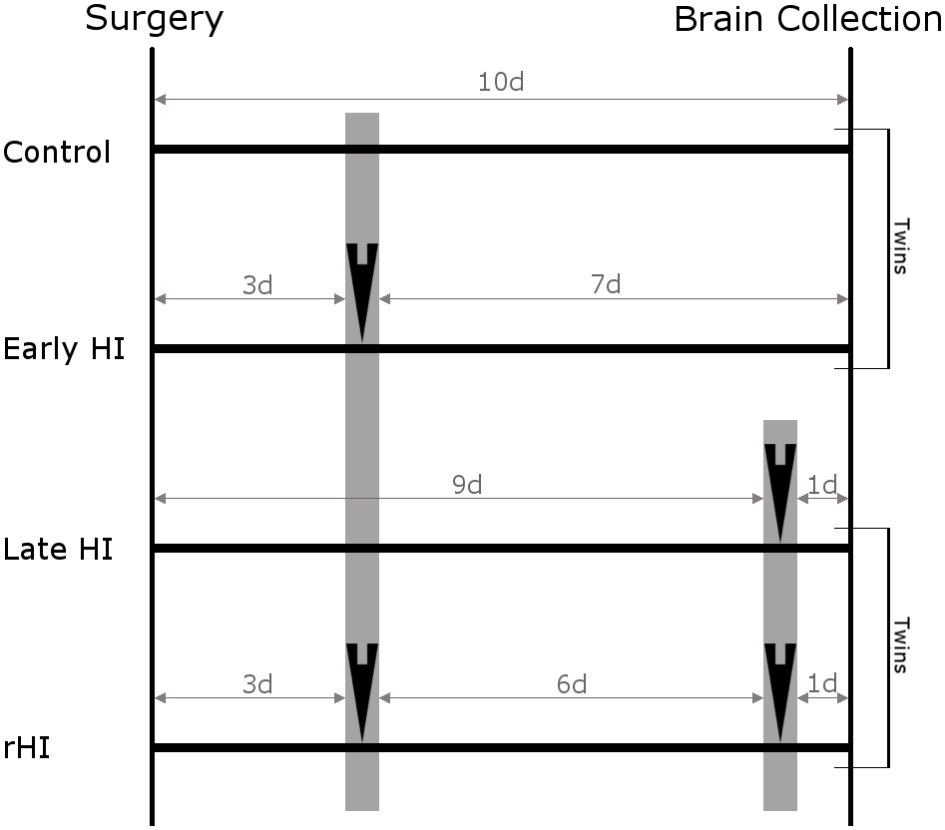
Schematic timeline of the ten day protocol for rHI studies showing the assignment of animals from twin pairs to the four experimental conditions. Animals recovered from surgery for 3 days before the initial exposure to hypoxemia with or without concurrent ischemia (black arrowheads indicate the timing of ischemia). The first gray shaded bar indicates that all groups sustained a 30-minute period of maternal hypoxemia at this time. Note that the twin control for the early HI group and the late HI twin of the rHI group did not sustain ischemia at this time. Thereafter, the early HI group and their twin controls survived for 7 days (i.e., 10 days after surgery). The rHI cases were exposed to a second episode of HI six days after the first HI episode. The corresponding twins for the rHI group were the late HI animals. The rHI group and the late HI cases were both exposed to an insult that involved maternal hypoxemia (gray shaded bar) and ischemia (arrowheads) at 24-hours before brain collection (i.e., 7 days after the initial insult and 10 days after surgery). Hence, the animals in all four groups survived for 10 days after surgery.

Moderate maternal and fetal hypoxemia was caused by lowering the inspired oxygen fraction of the ewe to 10.5% [19]. After 5 minutes of hypoxia, sustained cerebral hypoperfusion was initiated for 25 minutes by occlusion of the common brachiocephalic artery, during which time hypoxemia was maintained. As illustrated in figure 1, twin pairs of animals were assigned to either the control + early HI group or the late HI + rHI group. All animals recovered from surgery for at least 3 days prior to HI. Ewes assigned to the control + early HI group were subjected to ischemia (Fig. 1 black arrowheads) and hypoxemia (Fig. 1 gray bars) once and survived seven days before sacrifice. Ewes assigned to the late HI + rHI group sustained the HI protocol twice: once, seven days prior to sacrifice when the brachiocephalic artery of only one fetus was occluded (rHI), and again, one day prior to sacrifice when the brachiocephalic arteries of both fetuses were occluded.

In addition to the experimental design in figure 1, additional animals survived for 24 h, 2-weeks or 4-weeks after a single episode of HI and were subjected to a surgical and hypoxia-ischemia protocol identical to the early HI group. Hypoxia-ischemia protocols for the University of Auckland are described elsewhere [21].

### Physiological Monitoring

Prior to the start of the HI procedure, the catheters of each instrumented fetus were connected to pressure transducers and a digital chart recorder (PowerLab 16/30; ADInstruments, Sydney, Australia) to record pressure in the left fetal carotid artery relative to amniotic fluid pressure (mean arterial blood pressure). Fetal heart rate and blood pressure were calculated from triplicate measurements of the arterial pressure pulse intervals over a continuous recording of no less than 20 seconds (Table S1).

### Blood Analysis

Four blood samples (1 mL each) were collected anaerobically from the left fetal carotid artery during the course of the HI procedure. The first (baseline) sample was taken prior to the start of the study; the second (hypoxic) sample was 5 minutes after the start of the hypoxia; the third (hypoxia-ischemia) sample was 25 minutes after the start of the brachiocephalic artery occlusion; and the last (recovery) sample was 10 minutes after reversal of the HI by deflation of the occluder. All blood samples were analyzed for arterial pH, PO_2_, PCO_2_ corrected to 39°C, as well as hemoglobin content, glucose content, lactate content, oxygen saturation (SO_2_), and hematocrit (ABL725 blood gas analyzer; Radiometer Medical A/S, Bronshoj, Denmark; Table S1).

### Tissue Collection and Processing

Fetal brains were collected, weighed, and the cerebellum and brainstem removed by cutting the cerebral peduncles at the pontopeduncular junction. The remaining telencephalon and diencephalon was cut into five equivalent coronal blocks (<1 cm thick) in proportion to the distance between the frontal and occipital poles. The coronal blocks were immersed in 4% paraformaldehyde in 0.1 M phosphate buffer (pH 7.4, 4°C) for 3 to 5 days and stored at 4°C in phosphate-buffered saline (PBS) with 0.05% sodium azide (NaN_3_) [4]. Final one-week survival sample sizes: Control: N = 5; Early HI: N = 5; Late HI: N = 6; rHI: N = 6. Time course studies of HA and PH20 expression utilized tissue from animals from other ongoing studies that survived for 24 h (HI N = 3, Control N = 3), 2 weeks (HI N = 1, Control N = 1) or 4 weeks (HI N = 4, Control N = 1). During tissue harvest from 24 h survival fetuses, one prefrontal tissue block was rapidly frozen in liquid nitrogen for PCR analysis. Fetal ovine frontal white matter and testis for PCR and RNA-sequencing studies in Auckland, NZ were snap-frozen in liquid nitrogen.

### Immunohistochemistry

Coronal blocks containing the anterior (frontal) horn of the lateral ventricle were hemisected and one hemisphere was cut in the coronal plane using a vibrating microtome (VT1000S; Leica Microsystems Inc., Buffalo Grove, IL) into 50 µm thick serial sections and collected into cryoprotectant (30% v/v ethylene glycol, 15% m/v sucrose, 0.3 M PO_4_) before transfer to PBS for immunohistochemical analyses. Hyaluronic Acid (HA) was visualized with a biotinylated HA-binding protein (HABP; Seikagaku, Tokyo, Japan, 1∶200) followed by avidin-conjugated Rhodamine Red-X (Jackson ImmunoResearch, West Grove, PA, 1∶200) [6]. Frontal sections for preOL counts were double labeled with antibodies against O4 (Mouse IgM; 1∶500) and glial fibrillary acidic protein (GFAP) (Rabbit polyclonal, Dako, Carpinteria, CA, 1∶500) or activated caspase-3 (AC3; rabbit polyclonal, Cell Signaling, Danvers, MA, 1∶500) visualized with fluorescein isothiocyanate (FITC) (Mouse IgM, Vector Laboratories, Burlingame, CA, 1∶100) and Rhodamine Red-X (Rabbit IgG, Jackson ImmunoResearch, 1∶200) respectively [7], [14]. PH20 was visualized with a chicken polyclonal antiserum (1∶500; Aves Labs, Tigard, OR,) or a rabbit polyclonal antiserum (1∶400; gift from James Overstreet; University of California, Davis, Davis, CA), and visualized with FITC (Chicken or Rabbit IgG, both: Jackson ImmunoResearch, 1∶200). PH20 sections were co-labeled with GFAP (as above), visualized with Rhodamine Red-X. All sections were labeled with the nuclear counterstain Hoechst 33342 (Invitrogen/Life Technologies, Carlsbad, CA) to define anatomical boundaries.

Left-hemisphere coronal blocks containing the anterior (frontal) horn of the lateral ventricle were processed for paraffin embedding and serially sectioned at 6 µm. Paraffin sections were stained for GFAP (as above), ionized calcium-binding adapter molecule-1 (Iba-1; rabbit polyclonal, Vector Laboratories, 1∶200), activated caspase 3 (as above), oligodendrocyte transcription factor 2 (Olig2; Mouse monoclonal, gift from Dr. John Alberta: Dana-Farber Cancer Institute, Boston, MA; 1∶2,000), β-amyloid precursor protein (βAPP; MAB348, 1∶4,000; Millipore, Billerica, MA) or hematoxylin and eosin (H & E) [6]. All fluorescently-labeled sections (GFAP, Iba-1, AC3) were counterstained with Hoechst 33342 and visualized with a FITC secondary antibody (rabbit, Vector Laboratories, 1∶100) except for AC3 which was visualized with a Biotin-conjugated anti-rabbit secondary antibody (Jackson ImmunoResearch; 1∶200) and FITC-conjugated streptavidin (Jackson ImmunoResearch; 1∶400). Olig2 and a separate set of Iba-1 stained frontal sections were visualized with 3,3′-diaminobenzidine (DAB; Vector Laboratories). Parietal blocks containing thalamus were also paraffin-embedded, sectioned, and stained with Iba-1/DAB and H & E as described above.

### Quantification of White and Gray Matter Neuropathology and Neuroinflamation by H & E and Iba-1

Three near-adjacent paraffin sections (see above) were alternate stained for Iba-1 or H & E from a frontal or parietal block. We employed an ordinal rating scale to define the burden of WMI. The percentage of the white matter with necrosis was scored by a neuropathologist (MG) blinded to identifiers as follows: 0, no necrosis; 1, 1–25%; 2, 26–50%; 3, greater than 50%. Depending on survival time, necrosis was identified as diffuse pyknosis or loss of nuclear staining for the majority of cells in a region, dense infiltrates of activated microglia and macrophages, and/or cavitation. Severe pathology was defined as an average score of 2 or greater.

Gray matter injury was scored in frontal and parietal cerebral cortex and thalamus by a neuropathologist (MG) blinded to identifiers. For each brain region a composite ordinal score was generated based on review of tissue sections stained for H & E and Iba1/DAB, as described above for analysis of WMI. The 4-point scoring system was defined as: 0, normal; 1, focal injury; 2, multifocal patchy injury; 3, extensive injury or translaminar cortical necrosis.

### Definition of Frontal Periventricular White Matter Regions of Interest (ROIs)

All quantitative measures described below used the same ROI for the frontal periventricular white matter (PVWM) drawn in an unbiased manner by an investigator (MH) blinded to experimental conditions. Except were indicated, studies that analyzed fluorescently stained tissue sections employed ROIs whose boundaries were drawn using a Hoechst 33342 counterstain. The PVWM boundaries were defined by a horizontal line drawn tangent to the lateral ventricle at the head of the caudate nucleus, connecting to the fundi of adjacent sulci, and the cortical/white matter boundaries at those fundi [4].

### Quantification of GFAP and Iba-1 Area Fractions

WMI was assessed using an unbiased approach where lesions were not specifically selected. Using a motorized x, y stage mounted on an inverted fluorescent microscope (Leica DMIRE2, Leica Microsystems Inc., Buffalo Grove, IL), montages were generated from frontal tissue sections fluorescently stained for either GFAP or Iba-1. Montages were captured at 10X magnification (Leica, air, numerical aperture (na) = 0.25) using an Orca ER cooled CCD camera (Hamamatsu Photonics, Hamamatsu, Japan) and Stereo Investigator (SI, MBF Bioscience Inc., Williston, VT). To quantify the immunohistochemical staining, rolling-ball background subtraction with a 150-pixel radius was performed, and a pixel-intensity histogram was generated for the ROI using ImageJ (NIH, Bethesda, MD) and exported to R (R Foundation for Statistical Computing, Vienna, Austria). The peak of the histogram was calculated using the three highest frequency bins, the histogram curve integrated towards the background pixel side of the peak, and a value obtained for the area of this region. This area was then doubled to estimate the total distribution of background pixels in the image. The total background area was subtracted from the total region to define the GFAP or Iba-1 labeled area.

### Quantification of AC3 Positive Cells

The density of activated caspase-3 (AC3) was determined in frontal PVWM ROIs (ROIs determined as described above). Unbiased counts were acquired using the Meander Scan function of SI and a 63X Objective (Leica, air, na = 0.70).

### Quantification of Olig2 Positive Cells

For bright field imaging of Olig2, ROIs were defined as described above and gray-white matter boundaries were defined using Olig2. Ten randomly selected fields within the frontal PVWM ROI were imaged at 20X (Leica, air, na = 0.70). Images were exported to Image J and Olig2 positive nuclear profiles were manually counted using the program’s cell counter plugin.

### Quantification of O4-labeled Cells

Densities of intact and degenerating O4-labeled cells were estimated using high-precision design-based unbiased stereology using a Leica DMRA upright microscope with a motorized x, y stage. Contours of the frontal PVWM ROI were drawn as described above. With the SI optical fractionator probe, a digital sampling grid of 296.6×277.2 µm was laid over the entire ROI. Cells were counted by a single, blinded investigator (MH) in one section per animal with a 85×85 µm counting frame within each counting grid at 63X (HCX PL APO, oil, na = 1.4–0.6). The distance from the top of the section to the unbiased virtual counting zone was fixed at 3 µm (guard zone), and the height of the unbiased virtual counting zone (optical dissector) was set at 25 µm. Section thickness was measured at every fifth site. The inclusion criteria were any sharply focused part of soma (i.e., not processes). Intact and degenerating cells were defined by nuclear morphology. Schaffer coefficients of error (CE) were calculated.

### PH20 and HABP Image Acquisition

Images of PH20 and HABP staining were collected as single planes or as Z-stacks using a Nikon Confocal Microscope A1 system (Nikon Corporation, Tokyo, Japan). Stacks were acquired at either 20X (PLAN APO, Nikon, na = 0.8, step size = 1 µm), or 60X (PLAN APO, Nikon, na = 1.4, step size = 0.2 µm). All images were collected using a 17.9 µm pinhole radius, 561 nm and 405 nm solid state lasers, and an argon/krypton laser tuned to 488 nm.

### RNA extraction and RT-PCR

Tissues from testis and brain (white matter and cerebral cortex) from fetal sheep at 92–100 dGa were rapidly collected, snap frozen in liquid nitrogen, and stored at −80°C until use. Total RNA was extracted with TRIzol Reagent (Life Technologies, Auckland, NZ) and subject to DNase digestion (1 unit DNAse1 per 2 µg RNA; amplification grade, Sigma-Aldrich, Auckland, NZ) to eliminate genomic DNA contamination. First strand cDNA was synthesized using the SuperScript III First-Strand Synthesis System SuperMix (Life Technologies) with 2 µg of total RNA and the sheep SPAM1 gene-specific primer (5′-CTTGGCTGCACATTTTGGCT-3′), according to the manufacturer’s instructions. Controls were performed without reverse transcriptase (-RT). PCR was performed with Phire Hot Start II DNA Polymerase (Thermo Scientific, Auckland, NZ) using oligonucleotide primers designed against sheep SPAM1: FP: 5′-TCGTGTCCAGGAAGCCATTC-3′; RP: 5′-CTTGGCTGCACATTTTGGCT-3′ (designed to cross intron/exon boundary; 296 bp amplicon). PCR controls were also performed with PCR clean water as the template. All reactions were performed at 95°C for 3 min, followed by 40 cycles of incubation at 94°C for 15 s, 65°C for 30 s and 72°C for 30 s, followed by a final step at 72°C for 3 min. The expected size PCR products were analyzed on a 1.5% agarose gel with SYBR safe fluorescence detection and purified using the GenElute Gel Extraction kit (Sigma-Aldrich). Purified PCR products were then subject to a second round of nested PCR amplification using new sheep SPAM1 primers (FP: 5′-AGCGAGTGTTGAAAGTCCACT-3′; RP: 5′-CCAGAGGCACCTAGAGCAAC-3′; designed to cross intron/exon boundary; 132 bp amplicon) at 95°C for 3 min, followed by 40 cycles of incubation at 94°C for 15 s, 57°C for 30 s and 72°C for 30 s, followed by a final step at 72°C for 3 min. The expected size PCR products were analyzed on 1.5% agarose gel, purified and verified by automated DNA sequencing with an ABI Prism 377 sequencer (DNA Sequencing Facility, SBS, University of Auckland).

### RNA Sequencing (RNA-Seq)

The transcriptome from fetal ovine white matter at 24 h after sham or HI was determined. Total RNA was extracted as described above for RT-PCR. mRNA libraries were prepared from total RNA using Illumina TruSeq mRNA library (with ribodepletion) and sequenced on an Illumina HiSeq 2500 (150 million 2×100 bp PE reads). Run quality was assessed using SolexaQA and FastQC [23], [24]. Adaptors were removed using fastq-mcf [25] and reads below a PHRED score of 20 (Sanger FASTQ format) were discarded. For all samples (5 biological repeats of the sham and 7 repeats of the HI group), reads were mapped onto the oviAri3.1 sheep genome [25], using Tophat v.2.0.12 [26].

Following read mapping, transcripts were reassembled using the Cufflinks v2.2.1 workflow [27]. Both the EnsGene [28] and Genscan [29] predictions from the UCSC genome browser [30] were used to guide the assembly, and fragment bias correction and multiple read correction was used where possible. Both expression (within treatment) and differential expression (between treatments) were assessed using Cuffnorm v.2.2.1 and Cuffdiff v.2.2.1. Cross-replicate dispersion was assessed individually for each condition and the geometric method was used for library normalization. Mapping results and transcript reassembly were visualized in IGV [31] and expression and differential expression was analyzed using the R package cummeRbund [32].

### Statistical methods

Data analysis was performed using R software version 3.0.3. Comparisons across treatment groups were performed using the Kruskal-Wallis rank sum test with post-hoc multiple comparison Bonferroni-corrected Mann-Whitney U-tests for the majority of data, which were not normally distributed. In order to address the use of twins in our study design, repeated measures ANOVAs (RMA) were performed for each analysis across all four groups (GFAP AF, Iba-1 AF, Iba-1 scoring, H & E scoring, Olig2 densities, AC3 densities, O4+ densities) giving R^2^ for the ewe error term near zero in many cases and p>0.1 in all cases but one. We therefore employed nonparametric testing, which does not account for the ewe from which the fetus derived. Case-by-case correlations between multiple response variables were performed using Spearman’s rank correlation. Significance was defined as p<0.05.

## Results

### Recurrent HI results in a more severe spectrum of necrotic WMI

**Figure 2:**
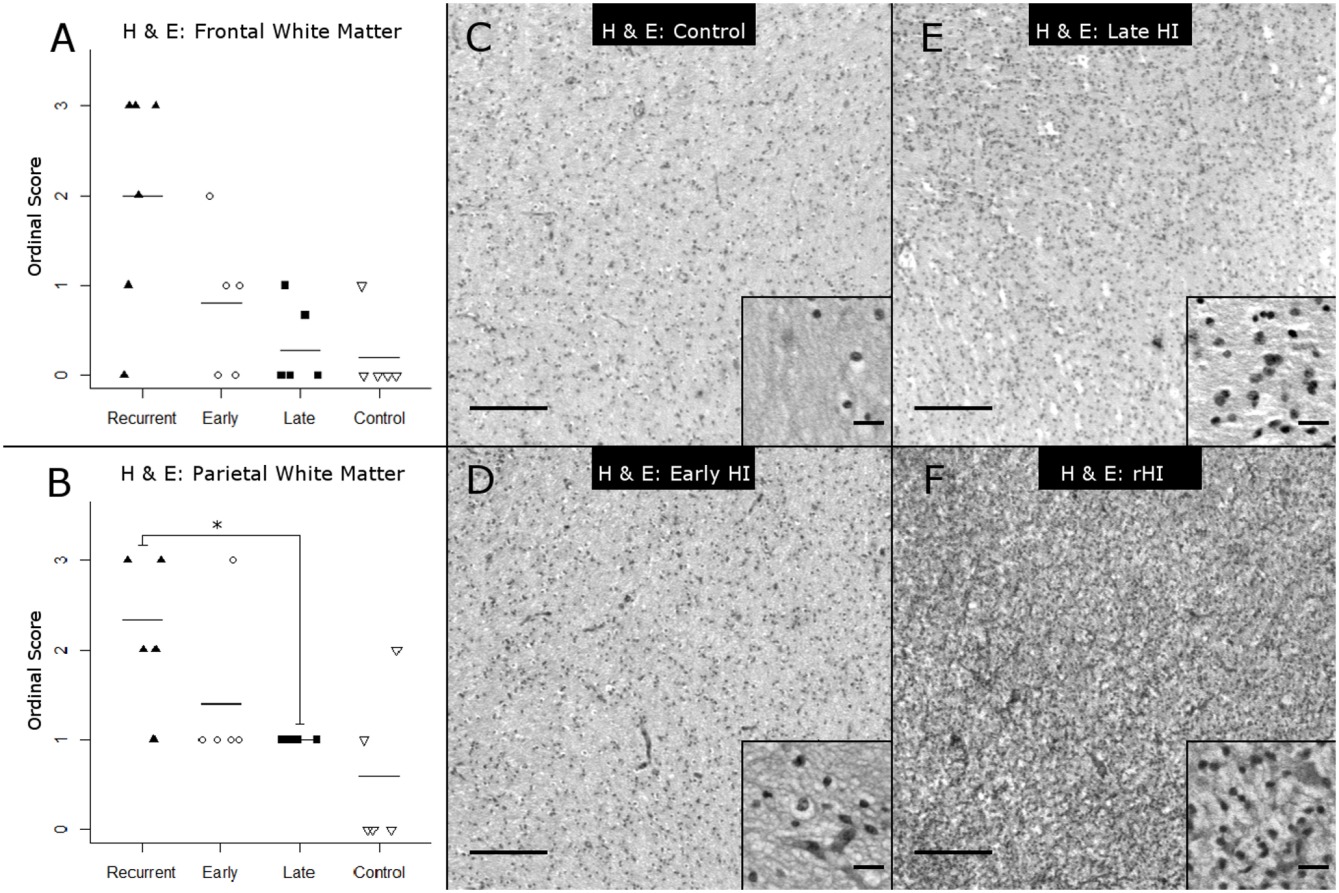
Spectrum of WMI in the four experimental conditions, as assessed by analysis of H & E staining using ordinal rating scores. (A) Neuropathological scoring of H & E staining in frontal white matter (Kruskal-Wallis H = 8.57, p = 0.036 on 3df; mean ± SD Control: 0.20±0.45; Late HI: 0.28±0.44; Early HI: 0.80±0.84; rHI: 2.0±1.3). (B) Neuropathological scoring of H & E stained parietal white matter (Kruskal-Wallis H = 10.5, p = 0.015; mean ± SD Control: 0.60±0.89; Late HI: 1.00±0.00; Early HI: 1.4±0.89; rHI: 2.3±0.82; Bonferroni-corrected post-hoc Mann-Whitney U-test: *rHI vs. Late HI, p = 0.036). (C–F) Representative images of H & E staining from (C) Control, (D) Early HI, (E) Late HI, (F) rHI frontal white matter. Panel scale bars: 200 µm; inset scale bars: 20 µm.

**Figure 3:**
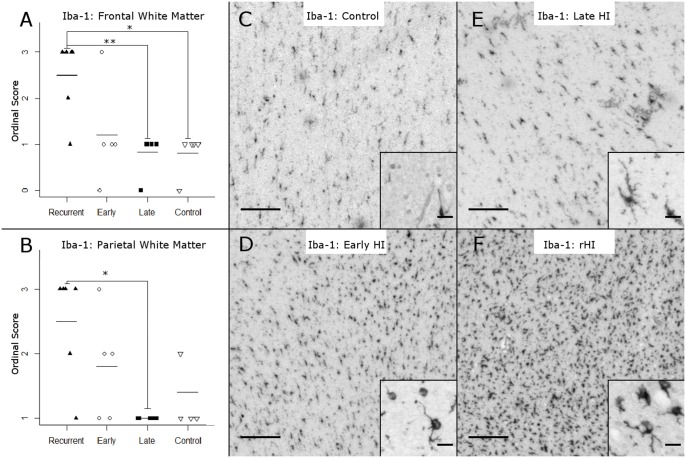
Spectrum of WMI in the four experimental conditions, as assessed by analysis of Iba-1 staining using ordinal rating scores. (A) Neuropathological scoring of Iba-1 staining in frontal white matter (Kruskal-Wallis H = 10.41, p = 0.015 on 3df; mean ± SD Control: 0.80±0.45; Late HI: 0.83±0.41; Early HI: 1.20±1.10; rHI: 2.50±0.84; Bonferroni-corrected post-hoc Mann-Whitney U-tests: *rHI vs. Control, p = 0.016; **rHI vs. Late HI, p = 0.01). (B) Neuropathological scoring of Iba-1 staining in parietal white matter (Kruskal-Wallis: H = 10.02, p = 0.018, on 3df, mean ± SD Control: 1.4±0.55; Late HI: 1.0±0.00; Early HI: 1.8±0.84; rHI: 2.5±0.84; Bonferroni-corrected post-hoc Mann-Whitney U-tests: *rHI vs. Late HI, p = 0.033). (C–F) Representative images of Iba-1 staining from (C) Control, (D) Early HI, (E) Late HI, (F) rHI frontal white matter. Panel scale bars: 200 µm, inset scale bars: 20 µm.

To define the response to rHI relative to single episodes of HI (Fig. 1), we employed several complementary approaches to quantify the severity of WMI in two regions of particular predilection for injury: the frontal and parietal cerebral white matter. We first employed an ordinal rating scale to estimate the severity of WMI based upon a neuropathological review of tissue sections stained with H & E (Fig. 2) or Iba-1 (Fig. 3). H & E staining detected a more severe spectrum of frontal (Fig. 2A) and parietal (Fig. 2B) WMI in the rHI cases that was more heavily weighted toward greater necrosis. A majority of the animals with pathology scores of 2 or greater in frontal (80%) and parietal white matter (71%) were in the rHI group (Fig. 2A, B). The rHI group was also associated with increased cellularity (Fig. 2C–F). Consistent with these findings, there was a significant increase in the number of frontal and parietal macroscopic foci of necrosis in the rHI group relative to the other treatment groups (p = 0.026; Kruskal-Wallis; Bonferroni-corrected post-hoc Mann-Whitney U-test showed a significant increase in the *rHI group vs. late HI group, p = 0.045).

Iba-1 staining showed a similar spectrum of WMI. A majority of the animals with pathology scores of 2 or greater in frontal (83%) and parietal white matter (50%) were in the rHI group (Fig. 3A, B). There was a significant increase in frontal WMI severity scores (Fig. 3A) for the rHI group (p = 0.015; Kruskal-Wallis; Post-hoc test: **rHI vs. Control, p = 0.016; *rHI vs. late HI, p = 0.010). Similarly, there was a significant increase in parietal WMI severity scores (Fig. 3B) for the rHI group (p = 0.018; Kruskal-Wallis; Post-hoc test: *rHI vs. late HI, p = 0.033).

**Figure 4:**
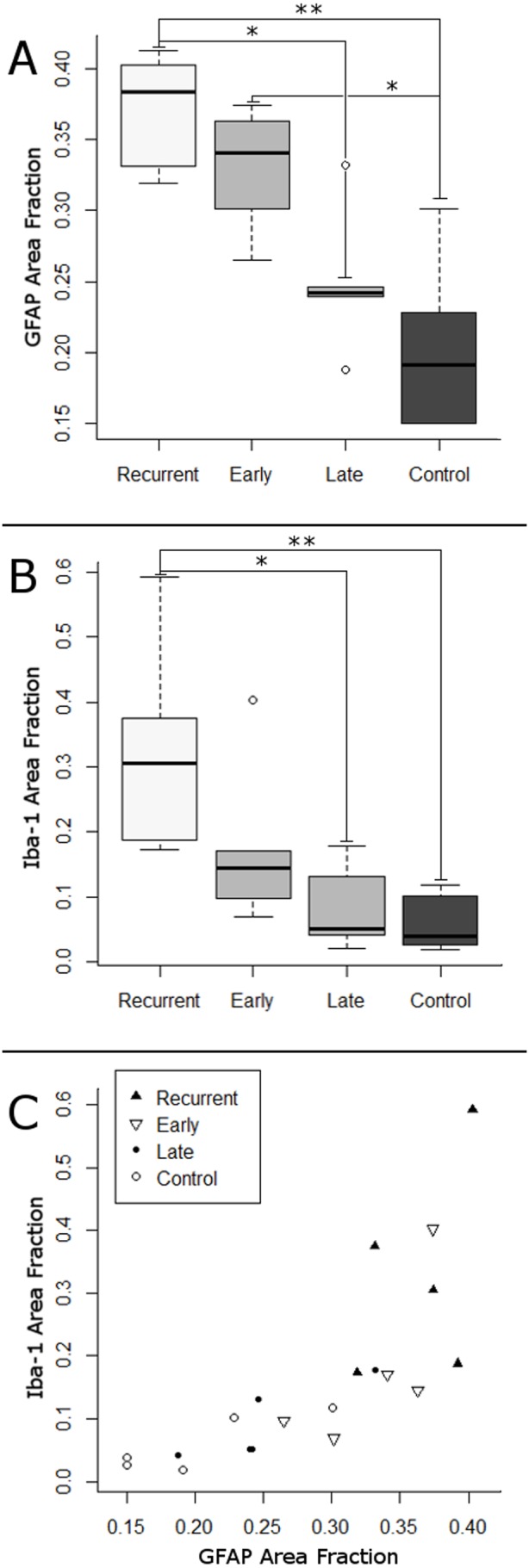
Spectrum of WMI in the four experimental conditions, as assessed by analysis of GFAP and Iba-1 staining using quantitation of area fractions stained for each marker. (A) Astrogliosis, measured by GFAP area fraction (AF), is increased following rHI (Kruskal-Wallis H = 13.96, p = 0.003, on 3df; mean ± SD Control: 0.20±0.06; Late HI: 0.25±0.05; Early HI: 0.33±0.05; rHI: 0.37±0.04; Bonferroni-corrected post-hoc Mann-Whitney U-tests: **rHI vs. Control, p = 0.002; *Early HI vs. Control, p = 0.018; *rHI vs. Late HI, p = 0.021). (B) Iba-1 AF revealed similar patterns to Iba-1 pathology in Fig. 3 (Kruskal-Wallis H = 11.7, p = 0.008; mean ± SD Control: 0.06±0.05; Late HI: 0.08±0.06; Early HI: 0.18±0.13; rHI: 0.33±0.170; Bonferroni-corrected post-hoc Mann-Whitney U-tests: **rHI vs. Control p = 0.01; *rHI vs. Late HI p = 0.020). (C) Iba-1 and GFAP AFs are significantly associated over a broad spectrum of WMI (***Spearman’s Rank Correlation: ρ = 0.91, p<0.0001).

We next analyzed the response to rHI using an unbiased quantitative image analysis protocol that provided continuous histo-pathological outcome measures for reactive astrogliosis and microglial activation in frontal periventricular white matter (PVWM). There was a significant increase in astrogliosis after rHI relative to other groups (Fig. 4A; p = 0.003; Kruskal-Wallis; Bonferroni-corrected post-hoc Mann-Whitney U-test: **rHI vs. Control, p = 0.002; *rHI vs late HI, p = 0.021; *early HI vs. control, p = 0.018). Similarly, there was a significant increase in microglia/macrophage activation after rHI relative to the other groups (Fig. 4B; p = 0.008; Kruskal-Wallis; Bonferroni-corrected post-hoc Mann-Whitney U-test: **rHI vs. Control, p = 0.01; *rHI vs. late HI, p = 0.02). These unbiased estimates of WMI defined by quantification of the astrocytic and microglial/macrophage responses were significantly associated across the entire range of injury responses for all treatment conditions (Fig. 4C; Spearman’s Rank Correlation: ρ = 0.91, p<0.0001). Thus, both semi-quantitative and unbiased estimates of WMI supported that rHI resulted in a more severe spectrum of WMI that was more heavily weighted toward a greater burden of necrosis.

### The severity of WMI and gray matter injury are significantly associated

**Figure 5:**
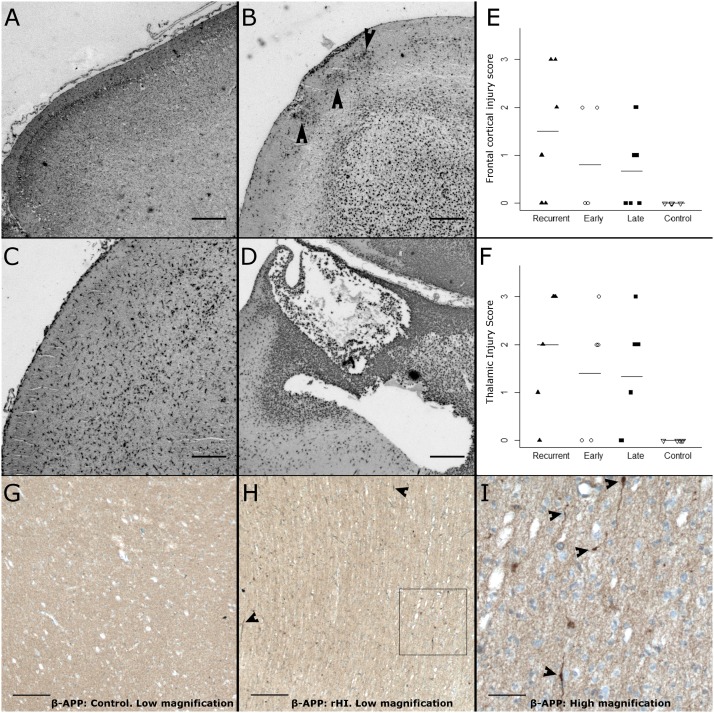
More severe WMI is associated with more severe gray matter injury. (A–D) Photomicrographs from four animals that sustained rHI that illustrate Iba1 staining used to define progressively more severe frontal cortical injury scores of 0 (A), 1 (B), 2 (C) and 3 (D), as defined in Materials and Methods. Note the variable cortical injury in the animals in B, C and D all of which sustained moderate-to-severe WMI. Note also the focal collections of activated microglia in B (arrowheads); the more diffuse distribution of activated microglia in C and the infiltrates of macrophages in a focal cystic necrotic cortical lesion in D. Scale bars A–D: 250 µm. (E, F) Neuropathological scoring of Iba-1 staining in frontal cortex (E; Kruskal-Wallis: H = 5.38, p = 0.146 on 3df; Mean ± SD Control: 0.00±0.00; Early HI: 0.80±1.10; Late HI: 0.67±0.82; rHI: 1.50±1.38) and thalamus (F; Kruskal-Wallis H = 7.32, p = 0.062 on 3df; Mean ± SD Control: 0.00±0.00; Early HI: 1.40±1.34; Late HI: 1.33±1.21; rHI: 2.00±1.26, Bonferroni-corrected post-hoc Mann-Whitney U-test: rHI vs. Control: p = 0.059). (G–I) Diffuse WMI was accompanied by a paucity of axonal degeneration as defined by staining for β-amyloid precursor protein (β-APP). (G) No degenerating axons were observed in control animals. (H) Representative image of the low levels of axonal degeneration observed in more severe diffuse WMI in an animal from the rHI group. This image from a rHI lesion shows rare degenerating axons (arrowheads) and within the inset box. (I) Detail of the inset shows the elevated staining for β-APP in several dystrophic-appearing axons (arrowheads) in this apparent small focus of necrosis. Scale bars (G–H) 100 µm; (I) 25 µm.

We next asked whether rHI was associated with more severe cortical or subcortical gray matter injury as defined by a semi-quantitative analysis of H & E or Iba1 staining. Figure 5A–D shows the representative spectrum of frontal cortical injury from which the ordinal rating scores were assigned. There were no significant differences in frontal, parietal or thalamic injury scores among the four groups. Figure 5E shows the data analyzed for frontal cortex (p = 0.15; Kruskal-Wallis), which was similar to parietal cortex (data not shown; p = 0.27; Kruskal-Wallis). We observed a near significant trend for greater injury in the thalamus in the rHI group vs. control (Fig. 5F; p = 0.062; Kruskal-Wallis; Bonferroni-corrected post-hoc Mann-Whitney U-test: rHI vs. Control, p = 0.059).

We next asked whether WMI severity was significantly associated with the severity of gray matter injury. We compared gray matter injury severity for both frontal and parietal cortex vs. WMI injury severity across all four groups of animals. There was a significant association between the severity of cortical injury and Iba1-defined WMI (Fig. 3A, B) at both the frontal (Spearman’s Rank Correlation ρ = 0.56, p = 0.007) and parietal levels (Spearman’s Rank Correlation ρ = 0.53, p = 0.011). When we compared H & E-defined WMI severity scores (Fig. 2A, B) with gray matter injury scores, we found similar significant associations at both the frontal (Spearman’s Rank Correlation ρ = 0.66, p<0.001) and parietal levels (Spearman’s Rank Correlation ρ = 0.06, p = 0.003). There were also significant associations between the severity of Iba-1-defined parietal WMI (Fig. 3B) and thalamic injury (Spearman’s Rank Correlation, ρ = 0.55, p = 0.008), and the severity of H & E-defined parietal WMI (Fig. 2B) and thalamic injury (Spearman’s Rank Correlation, ρ = 0.56, p = 0.006).

To analyze the relative susceptibility of axons in WMI among the four groups, we analyzed staining for the axonal injury marker beta-amyloid precursor protein (β-APP), which is a sensitive marker of degenerating neuro-axonal elements in the white matter in human WMI [6], [9] and in fetal ovine WMI [7]. Relative to control (Fig. 5G), we rarely detected β-APP-positive degenerating neuro-axonal elements within regions of diffuse WMI even in animals with more severe WMI and gray matter injury related to rHI (Fig. 5H, I).

### HA and the Hyaluronidase PH20 are elevated in HI lesions

**Figure 6:**
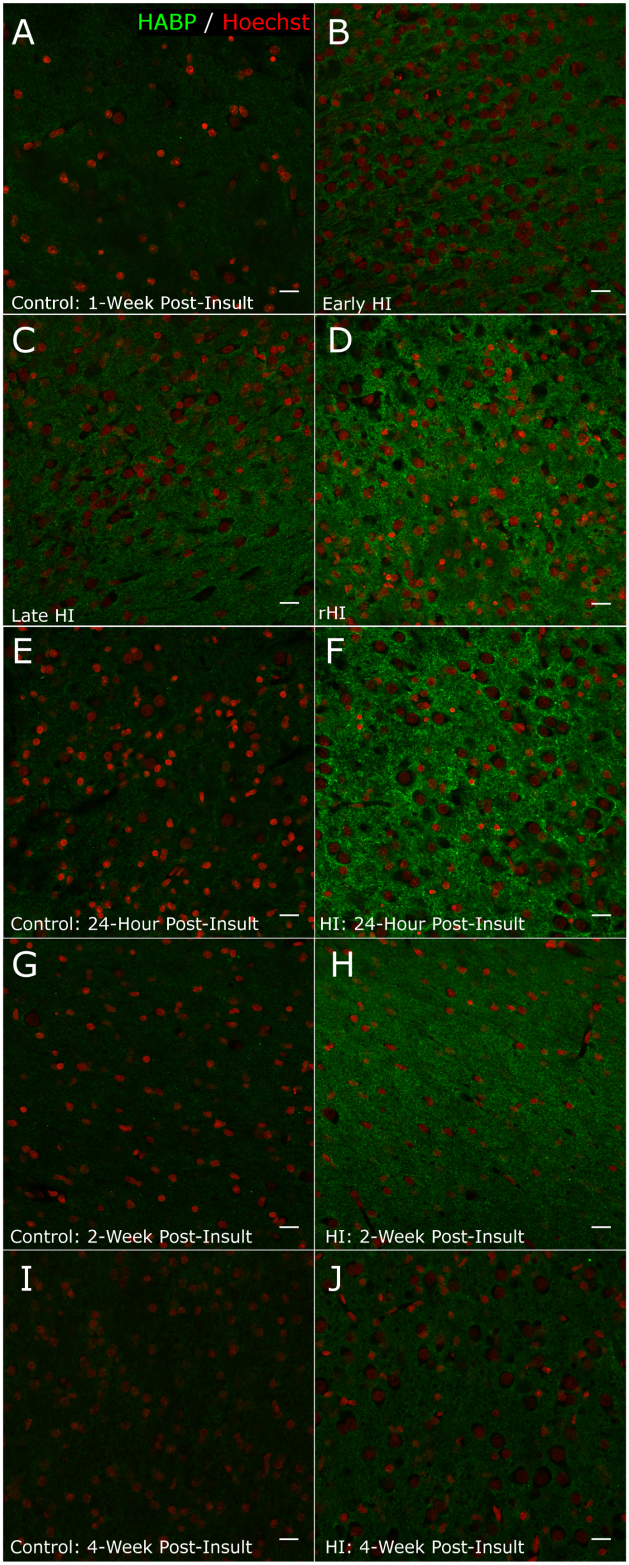
HA is present in fetal ovine white matter and displays a persistent increase for several weeks after HI. Representative confocal image of staining for HA with an HA binding protein (HABP) in frontal white matter (pseudocolors: green: HABP; red: Hoechst 33342-labeled nuclei). (A) Control: One-week post-insult. (B) Early HI. (C) Late HI. (D) rHI. (E) Control: 24-hours post-insult. (F) HI: 24-hours post-insult. (G) Control: Two weeks post-insult. (H) HI: Two weeks post-insult. (I) Control: Four weeks post-insult. (J) HI: Four weeks post-insult. Scale bars 20 µm.

Given the robust astrogliosis observed in white matter lesions, we next analyzed extracellular matrix molecules that are associated with disrupted maturation of OL progenitors in chronic WMI [3]. We previously found that HA levels are markedly elevated in the extracellular matrix in regions of reactive astrogliosis and that HA is a robust marker of chronic human preterm WMI [6]. We employed an HA binding protein to detect higher molecular weight forms of HA. We found a pronounced accumulation of HA in rHI lesions (Fig. 6D) relative to control (Fig. 6A). HA was also moderately increased in early HI (Fig. 6B) and late HI (Fig. 6C) WMI lesions. To further define the time course for HA to accumulate in response to HI, we analyzed WMI from a series of animals that had survived for 24 h, 2- or 4-weeks after HI. HA was found to robustly accumulate in WMI as early as 24 h after HI and to decline but remain elevated relative to control for at least 4 weeks (Fig. 6E–J).

**Figure 7:**
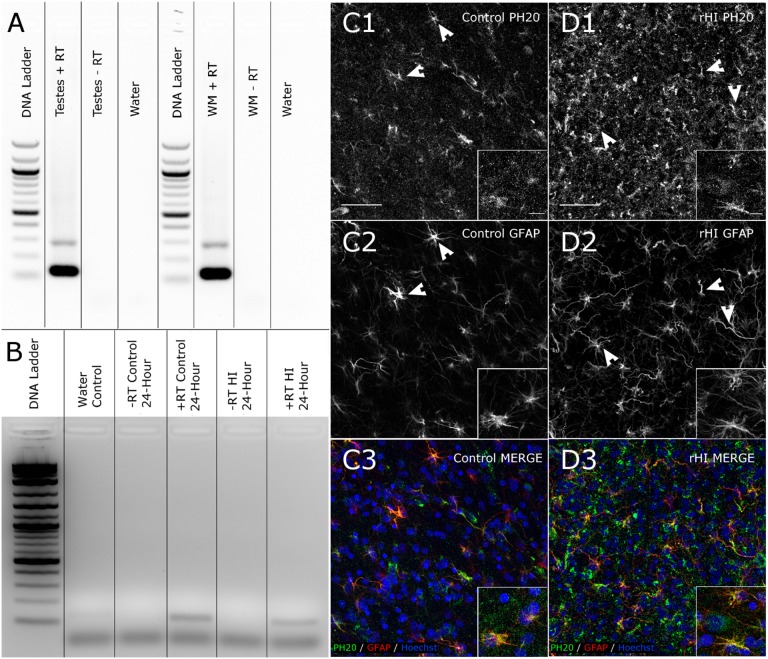
PH20 is present in fetal ovine white matter and shows enhancement following HI. (A) PH20 expression is detected in fetal ovine testis and white matter from New Zealand sheep (see Methods) by RT-PCR. (B) PH20 expression is detected in fetal ovine white matter from US sheep (see Methods) from controls and 24 h after HI by RT-PCR. (C–D) PH20 expression is detected by immunohistochemical staining in control white matter (C1) and displays enhanced staining after rHI (D1). Double-staining of PH20 with GFAP-labeled astrocytes in the same tissue sections shown in C1 and D1 (arrowheads). In D2, note the extensive reactive astrogliosis in response to rHI as compared to the astrocytes in control (C2). (C3, D3) Pseudocolor merge images demonstrate the extensive co-localization of PH20 and GFAP in astrocyte processes. (pseudocolors in C3 and D3: green: PH20; red: GFAP; blue: Hoechst 33342). Panel scale bars: 50 µm; inset scale bars: 10 µm.

We recently reported that a membrane-associated hyaluronidase, PH20 (SPAM1) is elevated in adult demyelinating multiple sclerosis lesions and digests high molecular weight forms of HA to a range of lower molecular weight HA fragments that block preOL maturation [18]. To determine if PH20 is also expressed in the fetal ovine brain, we employed a nested PCR approach using multiple primer sets for ovine PH20. PH20 expression was found in control fetal sheep (0.65 GA) in testes and in white matter. Sequencing of these 132 base pair bands confirmed that both PCR products were PH20 (Fig. 7A). In a separate experiment, PH20 expression was confirmed by RT-PCR in both the control and injured white matter at 24 h after HI (Fig. 7B).

We next employed RNA-Seq to confirm PH20 expression in tissue from controls (n = 4) and WMI at 24 h (n = 6). A putative sheep SPAM1 ortholog was identified using Genescan gene prediction, by homology to sheep RT-PCR sequencing results above, and by homology to SPAM1 genes from other species. This candidate (Genescan chr4.1745) showed expression in the sheep control white matter and in WMI at 24 h after HI as shown by reads mapping to the correct exons of the candidate gene, and this transcript was correctly reconstructed using the cufflinks workflow. Confirmation of RNA-Seq to reflect changes in our HI model was shown by a significant ∼2-fold increase in both GFAP mRNA (Genescan I.D. chr11.1380; q-value = 0.00096805) and CD44 mRNA (Genescan I.D. chr15.1539; q-value = 0.0358692) expression in WM lesions at 24 h recovery from HI.

Immunohistochemical staining for PH20 with a chicken antiserum against rat PH20 demonstrated that PH20 was expressed in control white matter (Fig. 7C1) and PH20 levels were increased in WMI from the rHI group (Fig. 7D1). Double staining for GFAP demonstrated that PH20 localized to astrocytes both in controls (Figs. 7C2, C3) and after WMI from rHI (Figs. 7D2, D3). Similar results were obtained with both the chicken (Fig. S1A, B) and a rabbit PH20 antiserum (Fig. S1C, D). PH20 staining was similarly detected in astrocytes in white matter lesions from the early HI (Fig. S1A) and late HI (Fig. S1B). PH20 staining was detected in astrocytes in white matter lesions by 24 h after HI vs. control (Fig. S1C, D) and robust PH20 staining persisted in astrocytes at 4 weeks after HI (Fig. S2).

### rHI stimulates greater expansion of the total pool of OL lineage cells

**Figure 8:**
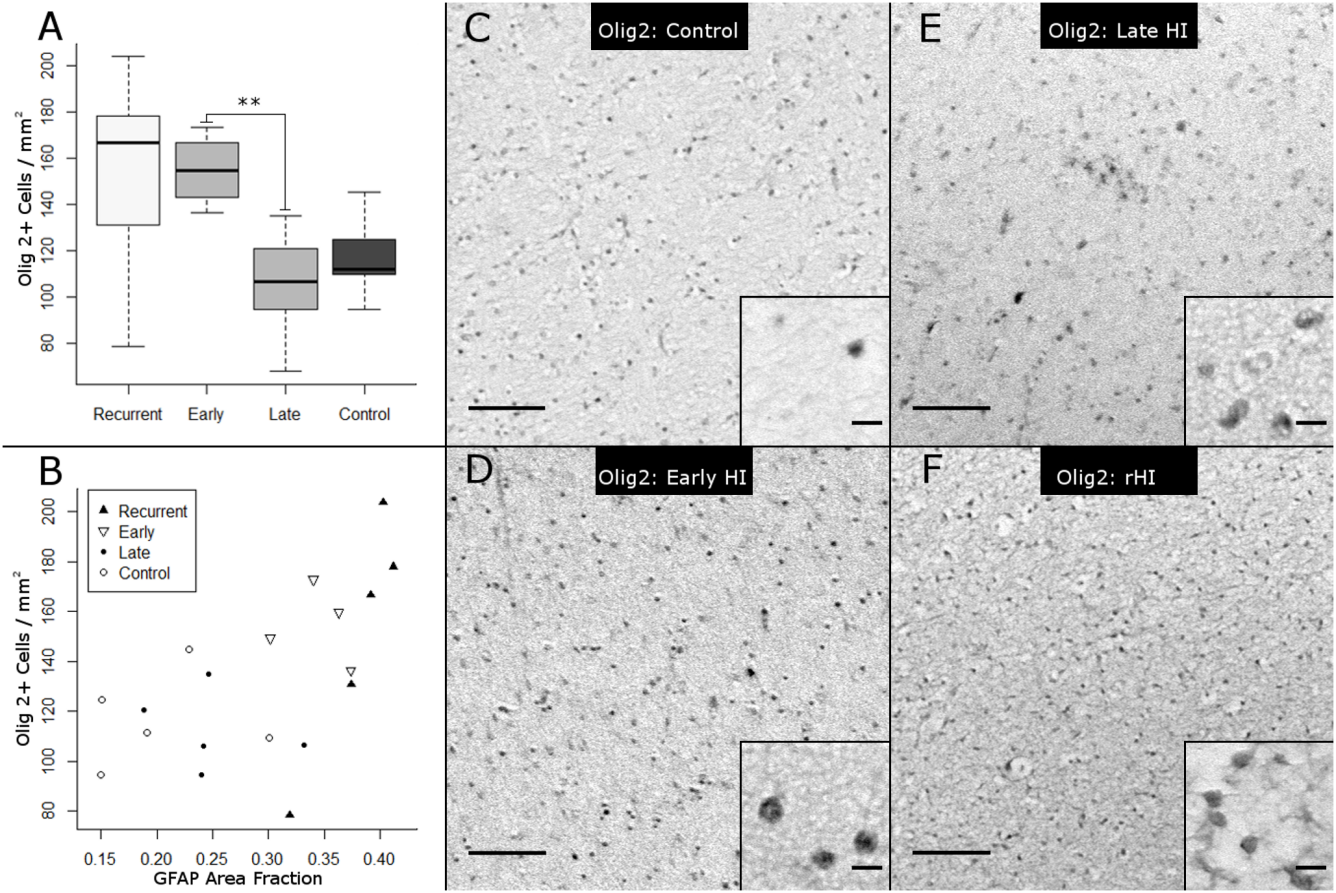
The total pool of OL lineage cells, defined by staining for Olig2, is increased in response to early HI. (A) Cell counts of Olig2 in the frontal PVWM for the four experimental conditions (Kruskal-Wallis H = 8.4, p = 0.038, on 3df; mean ± SD Control: 117.2±18.9 cells/mm^2^; Late HI: 105.1±23.0 cells/mm^2^; Early HI: 154.6±15.5 cells/mm^2^; rHI: 151.5±48.5 cells/mm^2^; Bonferroni-corrected post-hoc Mann-Whitney U-tests: **Early HI vs. Late HI: p = 0.008). (B) GFAP area fraction and Olig2 density are significantly associated over a broad spectrum of WMI (**Spearman’s Rank Correlation ρ = 0.63, p = 0.005). (C–F) Representative Olig2 images. (C) Control, (D) Early HI, (E) Late HI, (F) rHI. Panel scale bars: 100 µm. Inset scale bars: 10 µm.

Previous analysis of the response to an early HI insult demonstrated a progressive expansion of the total pool of OL lineage cells during a two-week period after fetal ovine HI [7]. We hypothesized that rHI would trigger a greater expansion of the OL pool in chronic WMI compared to a single episode of HI. Accordingly, we quantified the density of nuclei in the PVWM that labeled with Olig2, which labels all OL lineage stages (Figure 8). Olig-2-labeled cells were elevated in rHI and early HI cases relative to late HI and controls (Fig. 8A; p = 0.038, Kruskal-Wallis; Bonferroni-corrected post-hoc Mann-Whitney U-test: **early HI vs. late HI, p = 0.008).

The expansion of the total OL pool was significantly associated with several measures of WMI. Figure 8B shows a representative association between GFAP area fraction and the density of Olig2-labeled nuclei (Spearman’s rank correlation: ρ = 0.63, p = 0.005). Similarly, the density of Olig2-labeled nuclei was significantly associated with Iba-1 area fraction (Spearman: ρ = 0.54, p = 0.019). Thus, expansion of the OL pool was most pronounced in more severe chronic WMI. Representative images of Olig2 staining in control vs. the early HI, late HI and rHI groups are shown in Figures 8C–F, respectively.

### Expansion of the total OL pool is related to expansion of the pool of premyelinating OLs

**Figure 9:**
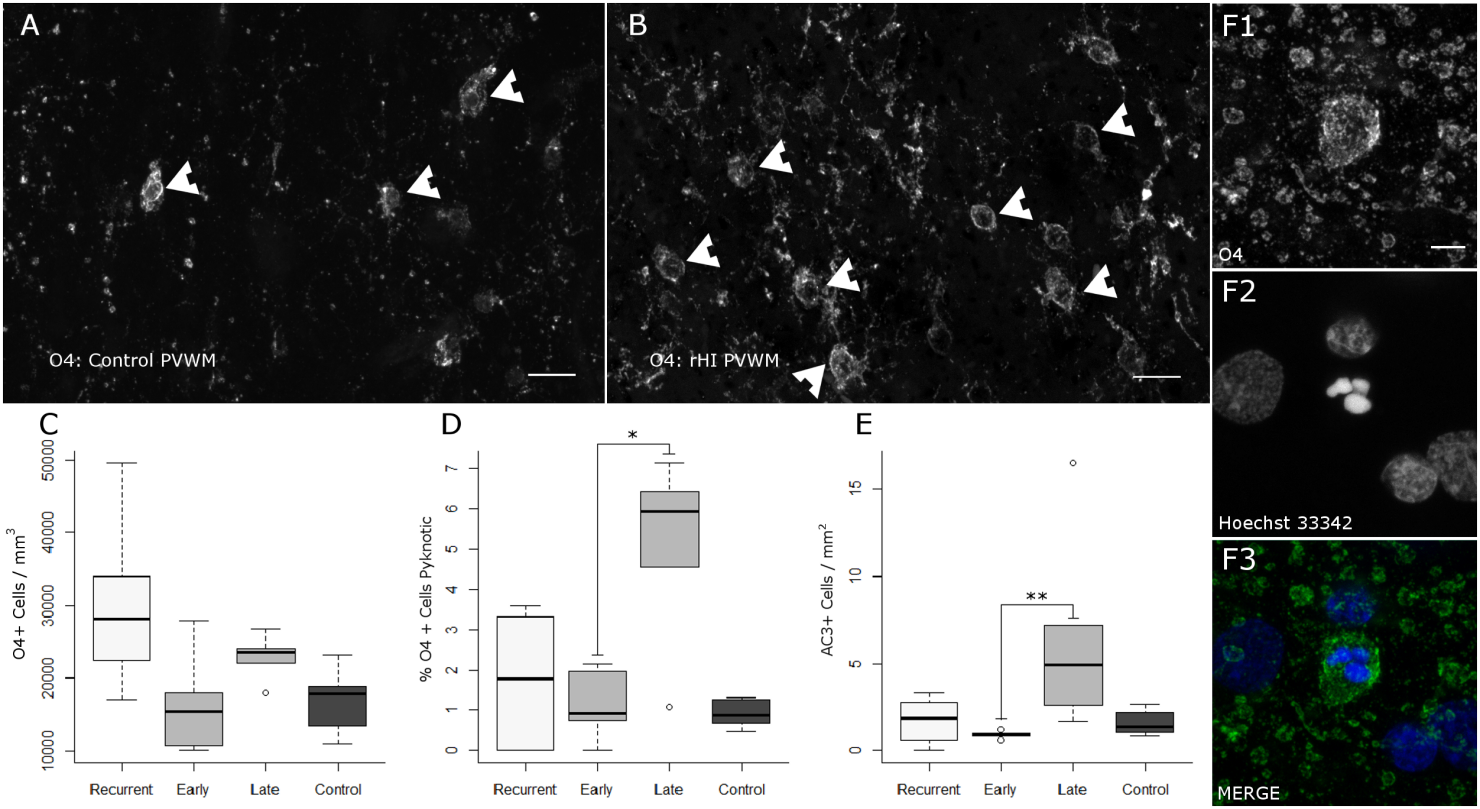
The pool of premyelinating OL lineage cells is significantly increased following rHI; PreOLs are less susceptible to acute degeneration following rHI than following late HI. (A–B) Representative confocal images of premyelinating OL lineage cells in the PVWM labeled with the O4 monoclonal antibody (arrowheads) in controls (A) and after rHI (B). Scale bars 20 µm. (C) Unbiased stereological counts of O4-labeled cells show an expansion of the preOL pool in the rHI group relative to control and early HI (Kruskal-Wallis: H = 7.7, p = 0.053, on 3 df; mean ± SD Control: 16,854±4,752 cells/mm^3^; Early HI: 16,430±7,182 cells/mm^3^; Late HI: 22,855±3,210 cells/mm^3^; rHI: 29,917±11,254 cells/mm^3^; CE range: 0.048–0.154). (D) Stereology further shows a decreased rate of preOL death following rHI relative to late HI (Kruskal-Wallis: H = 7.5, p = 0.057, on 3 df; mean ± SD Control: 0.9±0.4%; Early HI: 1.2±0.9%; Late HI: 5.0±2.4%; rHI: 1.7±1.8%; Bonferroni-corrected post-hoc Mann-Whitney U-tests: *Late HI vs. Control, p = 0.037; CE range for pyknotic counts: 0.21–0.95). (E) There is a greater density of activated caspase 3-labeled cells following a single late HI episode (Kruskal-Wallis H = 10.5, p = 0.015, on 3df; mean ± SD, Control: 1.62±0.76 Cells/mm^2^; Late HI: 6.30±5.40; Early HI: 0.92±0.21 cells/mm^2^; rHI: 1.73±1.33 cells/mm^2^; Bonferroni-corrected post-hoc Mann-Whitney U-tests: **Early HI vs. Late HI, p = 0.001). (F) Representative confocal images of a degenerating O4-positive cell with a fragmented pyknotic nucleus in a late HI case. (F1) O4, (F2) Hoechst 33342 nuclear stain, (F3) Merge. Scale bar: 5 µm.

Previous analysis of the response to an early HI insult demonstrated that the progressive expansion of the total pool of OL lineage cells was specifically related to an increase in the density of pre-myelinating preOLs [7], [14]. To determine if a similar response was observed after rHI, we analyzed the response of pre-myelinating cells labeled with the O4 monoclonal antibody. The density of O4-labeled cells throughout the PVWM appeared to be markedly lower in controls (Fig. 9A) relative to the rHI group (Fig. 9B). To quantify this response, we employed unbiased stereology, which was consistent with an increase in O4-labeled cells in response to rHI (Fig. 9C; p = 0.053, Kruskal-Wallis).

### Degeneration of premyelinating OLs is reduced after rHI relative to late HI

We previously observed that degeneration of premyelinating cells in neonatal rodents was markedly increased in response to rHI relative to an early HI insult [14]. We quantified the density of degenerating O4+ cells using the same stereological probes reported in Fig. 9C. The degeneration of O4-labeled cells was significantly elevated in the late HI group relative to control (Fig. 9D; p = 0.057 Kruskal-Wallis; Bonferroni-corrected post-hoc Mann-Whitney U-test: *Late HI vs. Control p = 0.037), in contrast to the rHI group. Consistent with these findings, there was a modest increase in the density of cells that stained for activated caspase 3 (AC3) in the PVWM of the late HI cases relative to the early HI group (Fig. 9E; p = 0.01 Kruskal-Wallis; Bonferroni-corrected post-hoc Mann-Whitney U-test: **Late HI vs. Early HI p = 0.001). These degenerating O4 labeled cells also displayed fragmented pyknotic nuclei by Hoechst 33342 nuclear counterstain (Fig. 9F). Double-labeling for O4 and AC3 (Fig. S3) similarly confirmed previous observations [4], [33] that the AC3 staining throughout the white matter localized to a low number of O4-labeled cells and other cell types were rarely AC3-positive.

## Discussion

Although WMI is the major cause of cerebral palsy (CP) in survivors of premature birth, the factors that contribute to more severe WMI have remained poorly defined. Despite continuing advances in neonatal care that have resulted in a less severe spectrum of WMI, a substantial number of patients continue to sustain cystic necrotic WMI or microscopic necrosis that is associated with significantly higher neurological morbidity [13]. We found that rHI predisposes the developing brain to a spectrum of WMI that is more heavily weighted toward necrosis, but with a reduced susceptibility of preOLs to recurrent degeneration. Our findings further support the notion that the incidence of more severe CP is related to preterm neonatal factors related to HI. WMI severity increases as the duration of a single episode of HI is prolonged [4]. In the present study, we employed a moderate HI protocol that after a single insult, reproducibly causes chronic diffuse WMI with a very low burden of microscopic necrosis similar to that which we have observed previously with an ischemia-only model [4], [7]. This diffuse WMI also resembles the injury commonly seen in contemporary human cohorts [6]. The WMI that resulted from a recurrence of this moderate HI insult was shifted toward a greater burden of cystic necrotic (i.e., macroscopic) WMI. This necrotic WMI resembles the lesions of periventricular leukomalacia (PVL) [8], [34], which were previously very common. Recurrent HI may predispose the developing brain to more severe WMI through multiple physiological mechanisms. We recently found that hypoxemia and hypotension both interact with hypoglycemia to bias toward more severe WMI [19]. Lower basal serum glucose levels were much more significantly associated with more severe WMI than were the magnitudes of hypoxemia or hypotension, which suggested a central role for energy failure in the shift to more severe injury.

Because there are no widely accepted markers of necrosis, we employed several complementary approaches to define the burden of necrotic WMI in each animal. We utilized a routine histopathological approach with H & E stained sections to estimate the burden of necrosis. Consistent with our recent findings [19], H & E staining appeared to be relatively less sensitive to a wide range of moderate levels of WMI when compared to Iba-1, as a marker of microglial and macrophage activation. Estimates of microglial and macrophage activation from ordinal scores appeared to agree more closely with unbiased quantitative measures of astrogliosis and microglial activation. Thus, commonly used pathological rating scales based on H & E are likely to underestimate moderate inflammatory WMI.

Alterations in the composition of the extracellular matrix were recently defined as robust markers of human chronic preterm WMI [6]. HA and one of its putative CNS receptors CD44 was highly enriched in human diffuse WMI defined by reactive astrogliosis. CD44 was an independent marker of WMI that was significantly associated with levels of GFAP across a broad spectrum of WMI. Although low levels of HA and PH20 were detected here in preterm fetal ovine white matter, no functional roles for HA in normal white matter development have been defined. It has been proposed that HA may play a role in maintaining the stem cell niche and holding committed progenitors in a less differentiated state [35]. Consistent with this notion, under pathological conditions of adult WMI, higher molecular weight forms of HA blocked preOL maturation [16], [17] via a mechanism that involved processing to smaller inhibitory HA digestion products [18]. We employed an HA binding protein that visualizes higher molecular weight forms of HA. With this approach, HA was found to rapidly increase by 24 hours after a single episode of fetal HI and to be elevated in response to rHI. After a single episode of HI, HA levels remained persistently elevated relative to controls but appeared to gradually decline by 4 weeks after HI.

This gradual decline in HA at later times after HI may reflect the activities of PH20 or other hyaluronidases as well as decreased HA synthesis by hyaluronan synthases [36]. In fact, PH20 was persistently expressed in white matter lesions by reactive astrocytes at 4 weeks after HI and in response to rHI. PH20 is highly enriched in sperm and has been widely studied for its roles in fertility and reproduction [37]–[39], but its expression in other organs including the CNS has been more difficult to detect. We employed multiple complementary approaches that support that PH20 is expressed in normal developing white matter. Using a nested RT-PCR approach, we independently detected a single PH20 transcript in white matter in two separate labs using tissue harvested from fetal sheep in New Zealand and the United States. This transcript was the same size as in fetal ovine testis, was obtained with multiple primer sets, did not originate from genomic DNA and was confirmed by sequencing to correspond to PH20. Consistent with these results, we detected PH20 in fetal ovine white matter by RNA-Seq in data from 10 animals and the fetal ovine PH20 sequence was highly homologous to rodent and human PH20. PH20 expression was also detected in fetal ovine WMI by RT-PCR and by RNA-Seq in tissue where GFAP and CD44 expression was increased 2–3 fold. PH20 was detected immunohistochemically in astrocytes using two separate polyclonal antisera from rabbit and chicken. The lower levels of PH20 staining in controls relative to the WMI groups support that PH20 expression is enhanced by WMI, consistent with recent results in adult rodents [18]. Future studies are needed to determine if PH20 plays a role in arrested preOL maturation and myelination failure in response to HI or rHI in fetal WMI. We did not observe arrested preOL maturation until 2 weeks after HI [7], which would require more extended survival than was feasible in the present study.

One unexpected result from this study was the apparent decreased susceptibility of preOLs to rHI in regions of non-necrotic WMI, despite enhanced susceptibility to white matter necrosis. PreOLs in preterm fetal sheep differed from the neonatal rat in their proliferative response to rHI. In the rat, rHI stimulated greater expansion in the total pool of OL lineage cells relative to single episodes of early or late HI [14]. By contrast, rHI and early HI caused a similar increase in the density of OL lineage cells (Fig. 8A). The expanded pool of OL lineage cells seen in the early HI and rHI groups may be related to a combination of early OL progenitor proliferation and recruitment, as previously observed for single episodes of HI in the neonatal rat [14] and the preterm fetal sheep [7]. At one week after single or recurrent HI, we did not detect evidence of cell proliferation using Ki67 staining (data not shown), which is consistent with our prior observations that the proliferative response after WMI is an acute response seen at 24 h after HI [7], [14]. We have similarly observed in chronic human WMI [6], that lesions had an expanded population of OL lineage cells, but Ki67 staining did not detect cell proliferation in these advanced lesions. The early cell proliferation response may be related to the elevated HA in acute lesions, which may play a role in expansion of the pool of OL lineage cells by blocking maturation of cells recruited to WMI.

Based upon our prior studies of rHI in neonatal rats [14], we expected but did not find that rHI would also markedly enhance preOL degeneration in fetal ovine WMI. In the rat, we observed that a single episode of HI on postnatal day 2 (P2) resulted in mostly caspase-3 independent apparent necrotic cell death [15]. However, rHI at P6 resulted in pronounced caspase-3-dependent apoptotic degeneration of almost all preOLs in white matter lesions [14]. The pronounced difference in response to rHI between the neonatal rat and the preterm fetal sheep may be multifactorial. One explanation for the pronounced apoptotic degeneration of preOLs in the rat may be a loss of trophic support from neuroaxonal elements that also diffusely degenerate in response to rHI. Rodents at P2 or P7 sustain extensive neuronal degeneration as a primary response to a single episode of HI [40], which is atypical for human preterm survivors with WMI [13], [33]. In fetal sheep, cortical neuronal degeneration was also typically less diffuse and severe than in rodents. Even in the more severely affected rHI group, we observed low levels of axonal degeneration in the white matter by β-APP staining. Hence, the more extensive preOL degeneration in response to rHI in rodents compared to fetal sheep may reflect greater loss of neuro-axonal trophic support.

Interestingly, preOL degeneration appeared greater after a single episode of late HI compared to rHI, and both conditions generated less preOL death than we previously observed for early HI [4]. This suggests the need for future studies to determine if rHI or the initial exposure to hypoxemia reduces preOL susceptibility to HI by a mechanism involving ischemic preconditioning. Although ischemic preconditioning has been demonstrated to protect against neuronal degeneration in several full term–equivalent neonatal rodent models of HI [41]–[44], no studies have been reported in preterm fetal sheep to determine if WMI can be similarly reduced. A recent study of hypoxic preconditioning in full term equivalent neonatal rats did not observe protection against preOL death [45], but few preOLs are present in the white matter at this time in development [46], [47]. This study did observe protection against myelin loss, which may be related to a reduction in neuroaxonal degeneration in this model [48]. We previously reported that arrested maturation of an expanded population of fetal ovine preOLs occurs after a single episode of HI [7]. Our present findings thus support that significant degeneration of this population of arrested preOLs does not occur following rHI and suggests that these cells may be a target to promote myelination in rHI lesions where HA and PH20 are enriched.

Several human autopsy studies of preterm brains have recently reported that neurons in both cortical and subcortical gray matter structures are susceptible to injury and loss [11], [12], [49]. We found that cortical and subcortical gray matter injury was generally mild to moderate in most animals after either single or recurrent HI episodes. Although we did not find that rHI was associated with significantly more severe gray matter injury, our findings support that the susceptibility of the gray matter to injury was strongly associated with the severity of WMI. Hence, severe cystic necrotic WMI was associated with more severe cortical and thalamic injury, which is consistent with the more severe gray matter pathological findings that have been reported in association with human cystic leukomalacia [50], [51].

The potential impact of rHI could only be partially evaluated by this study. In addition to the timing of rHI, other concurrent factors are likely to influence the response to rHI. In the clinical setting, other factors may influence the response to rHI that include complex systemic illness, nutritional, metabolic, endocrine, genetic and epigenetic factors. Postnatal infection is an important risk factor for WMI [52]–[54], and a number of inflammatory factors have been shown to cause WMI and promote preOL degeneration [55]–[57]. Recurrent postnatal infections, which may predispose to rHI, are associated with increased risk for progressive WMI in human preterm survivors [58]. The timing of rHI relative to the initial insult is also likely to be important. Our studies in fetal sheep support that the progression of preOL maturation arrest occurs more slowly than in rodents. A longer interval between HI events may predispose to greater diffuse WMI, since the pool of susceptible preOLs may be increased. A shorter interval between HI events may promote greater neuroprotection via mechanisms that involve ischemic tolerance. Despite the potential multifactorial nature of recurrent WMI, our data suggests the importance of aggressive treatment of preterm neonates who are at risk for rHI to avoid the potential for increased susceptibility to more severe white matter necrosis.

## Supporting Information

Figure S1 **PH20 co-localizes to the processes and soma of GFAP-labeled astrocytes and equivalent results are obtained with two antisera raised against PH20 in chicken (panels in A and B) and rabbit (panels in C and D).** Mostly punctate PH20 staining (A1–D1) co-localized (arrowheads) to GFAP-labeled astrocytes (A2–D2) in the (A) Early HI, (B) Late HI, (C) 24-hour survival control, (D) 24-hour HI groups. Pseudocolor merged images (A3–D3): green: PH20; red: GFAP; blue: Hoechst 33342-labeled nuclei. Scale bars: 10 µm.
(TIF)

Figure S2 **PH20 expression is persistently elevated in astrocytes four weeks after a single episode of HI at 93 dGa.** (A) 4-week survival control: PH20 (A1), GFAP (A2), and merge (A3). (B) 4-week survival HI: PH20 (B1), GFAP (B2), and merge (B3). Mostly punctate PH20 staining co-localized (arrowheads) to GFAP-labeled astrocytes. Scale bars: 20 µm.
(TIF)

Figure S3 **Typical appearance of activated caspase 3 (AC3; green) immunohistochemical staining in degenerating O4 antibody-labeled oligodendrocyte lineage cells (red) in control (A-panels), early HI (B-panels), late HI (C-panels) and rHI (D-panels).** The images illustrate the range of appearances of degenerating cells. Nuclear morphology is visualized with Hoechst 33342 fluorescent counterstain (blue). Some cells displayed a halo of few degenerating processes that were very fragmented (e.g., panels A3 and B3). Other cells displayed a complete loss of processes and were shrunken in appearance with condensed fragmented chromatin (e.g., panel C3). Cells at early stages of degeneration displayed numerous fragmented processes (e.g., panel D3) and nuclear morphology notable for multiple balls of condensed chromatin, as also supported by the appearance of the AC3 staining in D1.
(TIF)

Table S1 **Summary of Sheep Physiological Responses.** Physiological response to HI or rHI. Mean ± standard deviation. *p<0.05 within group vs. baseline (ANOVA or ANOVA on ranks if variances were unequal. Post hoc testing was Tukey as appropriate). ^‡^p<0.05 for a measure between groups (first and second HI were analyzed separately, using ANOVA/ANOVA on ranks with post hoc Tukey tests, and Student’s t-test respectively). **Indicates data could not be measured. ^†^Late HI fetuses were not rendered ischemic during the first HI procedure, explaining many of our differences between groups. Note that because control fetuses were not instrumented, data are not available for them; however, late HI fetuses were only exposed to hypoxemia during the first insult, which allowed the effect of hypoxemia to be assessed in isolation.
(PDF)

## References

[1] Back SA (2014) Cerebral white and gray matter injury in newborns: New insights into pathophysiology and management. Clin Perinatol 41: 1–24. 24524444 10.1016/j.clp.2013.11.001 PMC3947650

[2] Greisen G (2009) To autoregulate or not to autoregulate – that is no longer the question. Semin Pediatr Neurol 16: 207–215. 19945655 10.1016/j.spen.2009.09.002

[3] Back SA, Rosenberg PA (2014) Pathophysiology of glia in perinatal white matter injury. Glia 62: 1790–1815. 24687630 10.1002/glia.22658 PMC4163108

[4] Riddle A, Luo N, Manese M, Beardsley D, Green L, et al (2006) Spatial heterogeneity in oligodendrocyte lineage maturation and not cerebral blood flow predicts fetal ovine periventricular white matter injury. J Neurosci 26: 3045–3055. 16540583 10.1523/JNEUROSCI.5200-05.2006 PMC6673975

[5] McClure M, Riddle A, Manese M, Luo N, Rorvik D, et al (2008) Cerebral blood flow heterogeneity in preterm sheep: lack of physiological support for vascular boundary zones in fetal cerebral white matter. J Cereb Blood Flow Metab 28: 995–1008. 18091757 10.1038/sj.jcbfm.9600597 PMC3139427

[6] Buser J, Maire J, Riddle A, Gong X, Nguyen T, et al (2012) Arrested pre-oligodendrocyte maturation contributes to myelination failure in premature infants. Ann Neurol 71: 93–109. 22275256 10.1002/ana.22627 PMC3270934

[7] Riddle A, Dean J, Buser JR, Gong X, Maire J, et al (2011) Histopathological correlates of magnetic resonance imaging-defined chronic perinatal white matter injury. Ann Neurol 70: 493–507. 21796666 10.1002/ana.22501 PMC3170499

[8] Kinney H, Back S (1998) Human oligodendroglial development: relationship to periventricular leukomalacia. Semin Pediatr Neurol 5: 180–189. 9777676 10.1016/s1071-9091(98)80033-8

[9] Haynes RL, Billiards SS, Borenstein NS, Volpe JJ, Kinney HC (2008) Diffuse axonal injury in periventricular leukomalacia as determined by apoptotic marker fractin. Pediatr Res 63: 656–661. 18520330 10.1203/PDR.0b013e31816c825c PMC2770332

[10] Riddle A, Maire J, Gong X, Chen K, Kroenke C, et al (2012) Differential susceptibility to axonopathy in necrotic and non-necrotic perinatal white matter injury. Stroke 43: 178–184. 22076007 10.1161/STROKEAHA.111.632265 PMC3246543

[11] Andiman SE, Haynes RL, Trachtenberg FL, Billiards SS, Folkerth RD, et al (2010) The cerebral cortex overlying periventricular leukomalacia: analysis of pyramidal neurons. Brain Pathol 20: 803–814. 20331617 10.1111/j.1750-3639.2010.00380.x PMC2913678

[12] Kinney H, Haynes R, Xu G, Andiman S, Folkerth R, et al (2012) Neuron deficit in the white matter and subplate in periventricular leukomalacia. Ann Neurol 71: 397–406. 22451205 10.1002/ana.22612 PMC3315053

[13] Back S, Miller S (2014) Brain injury in premature neonates: A primary cerebral dysmaturation disorder? Ann Neurol 75: 469–486. 24615937 10.1002/ana.24132 PMC5989572

[14] Segovia K, McClure M, Moravec M, Luo N, Wang Y, et al (2008) Arrested oligodendrocyte lineage maturation in chronic perinatal white matter injury. Ann Neurol 63: 517–526. 10.1002/ana.21359 PMC3140464 18393269

[15] Back SA, Han BH, Luo NL, Chrichton CA, Tam J, et al (2002) Selective vulnerability of late oligodendrocyte progenitors to hypoxia-ischemia. J Neurosci 22: 455–463. 11784790 10.1523/JNEUROSCI.22-02-00455.2002 PMC6758669

[16] Back S, Tuohy T, Chen H, Wallingford N, Craig A, et al (2005) Hyaluronan accumulates in demyelinated lesions and inhibits oligodendrocyte progenitor maturation. Nat Med 9: 966–972. 10.1038/nm1279 16086023

[17] Sloane J, Batt C, Ma Y, Harris Z, Trapp B, et al (2010) Hyaluronan blocks oligodendrocyte progenitor maturation and remyelination through TLR2. Proc Natl Acad Sci USA 107: 11555–11560. 20534434 10.1073/pnas.1006496107 PMC2895128

[18] Preston M, Gong X, Su W, Matsumoto SG, Banine F, et al (2013) Digestion products of the PH20 hyaluronidase inhibit remyelination. Ann Neurol 73: 266–280. 23463525 10.1002/ana.23788 PMC3608752

[19] Riddle A, Maire J, Cai V, Nguyen T, Gong X, et al (2013) Hemodynamic and metabolic correlates of perinatal white matter injury severity. PLoS one 8: e82940. 24416093 10.1371/journal.pone.0082940 PMC3886849

[20] Back SA, Riddle A, Dean J, Hohimer AR (2012) The instrumented fetal sheep as a model of cerebral white matter injury in the premature infant. Neurotherapeutics 9: 359–370. 22399133 10.1007/s13311-012-0108-y PMC3337024

[21] Fraser M, Bennet L, Helliwell R, Wells S, Williams C, et al (2007) Regional specificity of magnetic resonance imaging and histopathology following cerebral ischemia in preterm fetal sheep. Reprod Sci 14: 182–191. 17636230 10.1177/1933719107299612

[22] Baldwin B, Bell F (1963) The anatomy of the cerebral circulation of the sheep and ox. The dynamic distribution of the blood supplied by the carotid and vertebral arteries to cranial regions. J Anat, Lond 97: 203–215. 13969329 PMC1244221

[23] Cox MP, Peterson DA, Biggs PJ (2010) SolexaQA: At-a-glance quality assessment of Illumina second-generation sequencing data. BMC Bioinformatics 11: 485. 20875133 10.1186/1471-2105-11-485 PMC2956736

[24] Andrews S (2012) “FastQC.” A quality control tool for high throughput sequence data. Available: http://wwwbioinformaticsbabrahamacuk/projects/fastqc/(accessed 8/4/14).

[25] Aronesty E (2011) ea-utils: “Command-line tools for processing biological sequencing data”. Available: http://codegooglecom/p/ea-utils (accessed 8/04/14).

[26] Kim D, Pertea G, Trapnell C, Pimentel H, Kelley R, et al (2013) TopHat2: accurate alignment of transcriptomes in the presence of insertions, deletions and gene fusions. Genome Biol 14: R36. 23618408 10.1186/gb-2013-14-4-r36 PMC4053844

[27] Trapnell C, Roberts A, Goff L, Pertea G, Kim D, et al (2012) Differential gene and transcript expression analysis of RNA-seq experiments with TopHat and Cufflinks. Nat Protoc 7: 562–578. 22383036 10.1038/nprot.2012.016 PMC3334321

[28] Hubbard T, Barker D, Birney E, Cameron G, Chen Y, et al (2002) The Ensembl genome database project. Nucleic Acids Res 30: 38–41. 11752248 10.1093/nar/30.1.38 PMC99161

[29] Burge C, Karlin S (1997) Prediction of complete gene structures in human genomic DNA. J Mol Biol 268: 78–94. 9149143 10.1006/jmbi.1997.0951

[30] Kent WJ, Sugnet CW, Furey TS, Roskin KM, Pringle TH, et al (2002) The human genome browser at UCSC. Genome Res 12: 996–1006. 12045153 10.1101/gr.229102 PMC186604

[31] Robinson JT, Thorvaldsdottir H, Winckler W, Guttman M, Lander ES, et al (2011) Integrative genomics viewer. Nat Biotechnol 29: 24–26. 21221095 10.1038/nbt.1754 PMC3346182

[32] Team RDC R: A language and environment for statistical computing. R Foundation for Statistical Computing, Vienna, Austria. Available: http://wwwR-projectorg/(accessed 8/04/14).

[33] Back SA, Luo NL, Mallinson RA, O’Malley JP, Wallen LD, et al (2005) Selective vulnerability of preterm white matter to oxidative damage defined by F_2_-isoprostanes. Ann Neurol 58: 108–120. 15984031 10.1002/ana.20530

[34] Banker B, Larroche J (1962) Periventricular leukomalacia of infancy. A form of neonatal anoxic encephalopathy. Arch Neurol 7: 386–410. 13966380 10.1001/archneur.1962.04210050022004

[35] Sherman L, Feistel K (2008) Hyaluronan as a regulatory component of neural stem cell and progenitor cell niches. Glycoforum: Science of Hyaluronan. Available: http://www.glycoforum.gr.jp/science/hyaluronan/HA30/HA30E.html.

[36] Sherman L, Back S (2008) A GAG reflex prevents repair of the damaged CNS. Trends Neurosci 31: 44–52. 18063497 10.1016/j.tins.2007.11.001

[37] Primakoff P, Lathrop W, Woolman L, Cowan A, Myles D (1988) Fully effective contraception in male and female guinea pigs immunized with the sperm protein PH-20. Nature 335: 543–546. 3419530 10.1038/335543a0

[38] Yudin AI, Vandevoort CA, Li MW, Overstreet JW (1999) PH-20 but not acrosin is involved in sperm penetration of the macaque zona pellucida. Mol Reprod Dev 53: 350–362. 10369396 10.1002/(SICI)1098-2795(199907)53:3<350::AID-MRD11>3.0.CO;2-9

[39] Kimura M, Kim E, Kang W, Yamashita M, Saigo M, et al (2009) Functional roles of mouse sperm hyaluronidases, HYAL5 and SPAM1, in fertilization. Biol Reprod 81: 939–947. 19605784 10.1095/biolreprod.109.078816

[40] Vannucci RC, Vannucci SJ (2005) Perinatal hypoxic-ischemic brain damage: evolution of an animal model. Dev Neurosci 27: 81–86. 16046840 10.1159/000085978

[41] Gidday JM, Fitzgibbons J, Shah A, Park TS (1994) Neuroprotection from ischemic brain injury by hypoxic preconditioning in the neonatal rat. Neurosci Lett 168: 221–224. 8028780 10.1016/0304-3940(94)90455-3

[42] Lin WY, Chang YC, Lee HT, Huang CC (2009) CREB activation in the rapid, intermediate, and delayed ischemic preconditioning against hypoxic-ischemia in neonatal rat. J Neurochem 108: 847–859. 19183266 10.1111/j.1471-4159.2008.05828.x

[43] Autheman D, Sheldon RA, Chaudhuri N, von Arx S, Siegenthaler C, et al (2012) Glutathione peroxidase overexpression causes aberrant ERK activation in neonatal mouse cortex after hypoxic preconditioning. Pediatr Res 72: 568–575. 23007029 10.1038/pr.2012.124 PMC3529181

[44] Ran R, Xu H, Lu A, Bernaudin M, Sharp FR (2005) Hypoxia preconditioning in the brain. Dev Neurosci 27: 87–92. 16046841 10.1159/000085979

[45] Suryana E, Jones NM (2014) The effects of hypoxic preconditioning on white matter damage following hypoxic-ischaemic injury in the neonatal rat brain. Int J Dev Neurosci 37C: 69–75. 10.1016/j.ijdevneu.2014.06.007 25009121

[46] Craig A, Ling Luo N, Beardsley DJ, Wingate-Pearse N, Walker DW, et al (2003) Quantitative analysis of perinatal rodent oligodendrocyte lineage progression and its correlation with human. Exp Neurol 181: 231–240. 12781996 10.1016/s0014-4886(03)00032-3

[47] Dean J, Moravec M, Grafe M, Abend N, Ren J, et al (2011) Strain-specific differences in perinatal rodent oligodendrocyte lineage progression and its correlation with human. Dev Neurosci 33: 251–260. 21865655 10.1159/000327242 PMC3225247

[48] Jones NM, Bergeron M (2001) Hypoxic preconditioning induces changes in HIF-1 target genes in neonatal rat brain. J Cereb Blood Flow Metab 21: 1105–1114. 11524615 10.1097/00004647-200109000-00008

[49] Pierson CR, Folkerth RD, Billiards SS, Trachtenberg FL, Drinkwater ME, et al (2007) Gray matter injury associated with periventricular leukomalacia in the premature infant. Acta Neuropathol 114: 619–631. 17912538 10.1007/s00401-007-0295-5 PMC2080348

[50] Marin-Padilla M (1997) Developmental neuropathology and impact of perinatal brain damage. II: white matter lesions of the neocortex. J Neuropathol Exp Neurol 56: 219–235. 9056536 10.1097/00005072-199703000-00001

[51] Marin-Padilla M (1999) Developmental neuropathology and impact of perinatal brain damage. III: gray matter lesions of the neocortex. J Neuropathol Exp Neurol 58: 407–429. 10331430 10.1097/00005072-199905000-00001

[52] Chau V, Brant R, Poskitt KJ, Tam EW, Synnes A, et al (2012) Postnatal infection is associated with widespread abnormalities of brain development in premature newborns. Pediatr Res 71: 274–279. 22278180 10.1038/pr.2011.40 PMC3940469

[53] Chau V, Poskitt KJ, McFadden DE, Bowen-Roberts T, Synnes A, et al (2009) Effect of chorioamnionitis on brain development and injury in premature newborns. Ann Neurol 66: 155–164. 19743455 10.1002/ana.21713

[54] Shah DK, Doyle LW, Anderson PJ, Bear M, Daley AJ, et al.. (2008) Adverse neurodevelopment in preterm infants with postnatal sepsis or necrotizing enterocolitis is mediated by white matter abnormalities on magnetic resonance imaging at term. J Pediatr 153: 170–175, 175 e171. 10.1016/j.jpeds.2008.02.033 18534228

[55] Fan L, Mitchell H, Tien L, Zheng B, Pang Y, et al (2008) alpha-Phenyl-n-tert-butyl-nitrone reduces lipopolysaccharide-induced white matter injury in the neonatal rat brain. Dev Neurobiol 68: 365–378. 18161853 10.1002/dneu.20591

[56] Favrais G, van de Looij Y, Fleiss B, Ramanantsoa N, Bonnin P, et al (2011) Systemic inflammation disrupts the developmental program of white matter. Ann Neurol 70: 550–565. 21796662 10.1002/ana.22489

[57] Dean J, van de Looij Y, Sizonenko S, Lodygensky G, Lazeyras F, et al (2011) Delayed Cortical Impairment Following Lipopolysaccharide Exposure in Preterm Fetal Sheep. Ann Neurol 70: 846–856. 22002627 10.1002/ana.22480

[58] Glass HC, Bonifacio SL, Chau V, Glidden D, Poskitt K, et al (2008) Recurrent postnatal infections are associated with progressive white matter injury in premature infants. Pediatrics 122: 299–305. 18676547 10.1542/peds.2007-2184

