# Supplementary figures and images for: Role of Recurrent Hypoxia-Ischemia in Preterm White Matter Injury Severity
Source: PLoS One. 2014 Nov 12;9(11):e112800. doi: 10.1371/journal.pone.0112800 (PMC4229227; doi:10.1371/journal.pone.0112800)

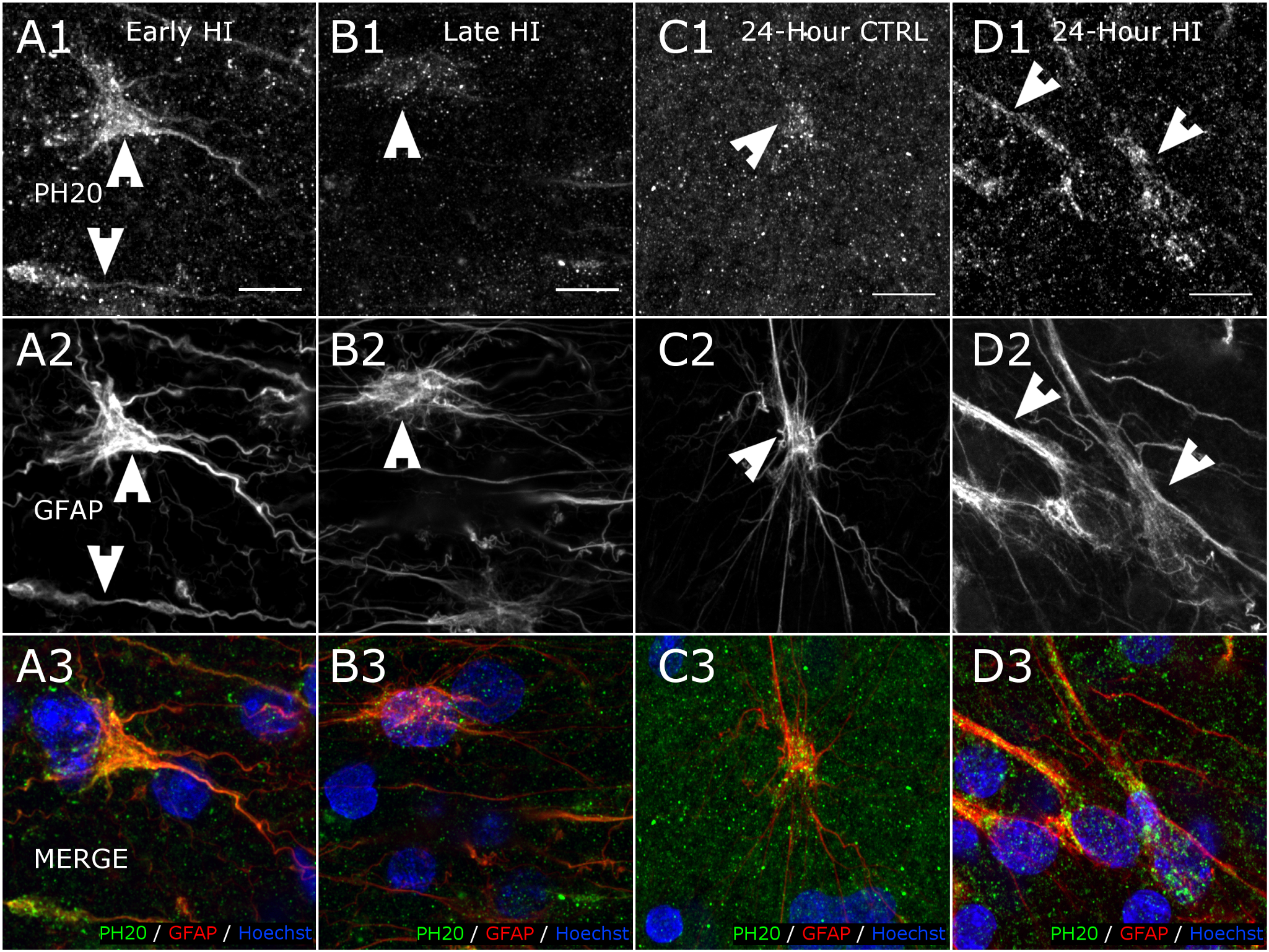

Supplement: Figure S1 — PH20 co-localizes to the processes and soma of GFAP-labeled astrocytes and equivalent results are obtained with two antisera raised against PH20 in chicken (panels in A and B) and rabbit (panels in C and D). Mostly punctate PH20 staining (A1–D1) co-localized (arrowheads) to GFAP-labeled astrocytes (A2–D2) in the (A) Early HI, (B) Late HI, (C) 24-hour survival control, (D) 24-hour HI groups. Pseudocolor merged images (A3–D3): green: PH20; red: GFAP; blue: Hoechst 33342-labeled nuclei. Scale bars: 10 µm. (TIF) [file pone.0112800.s001.tif]

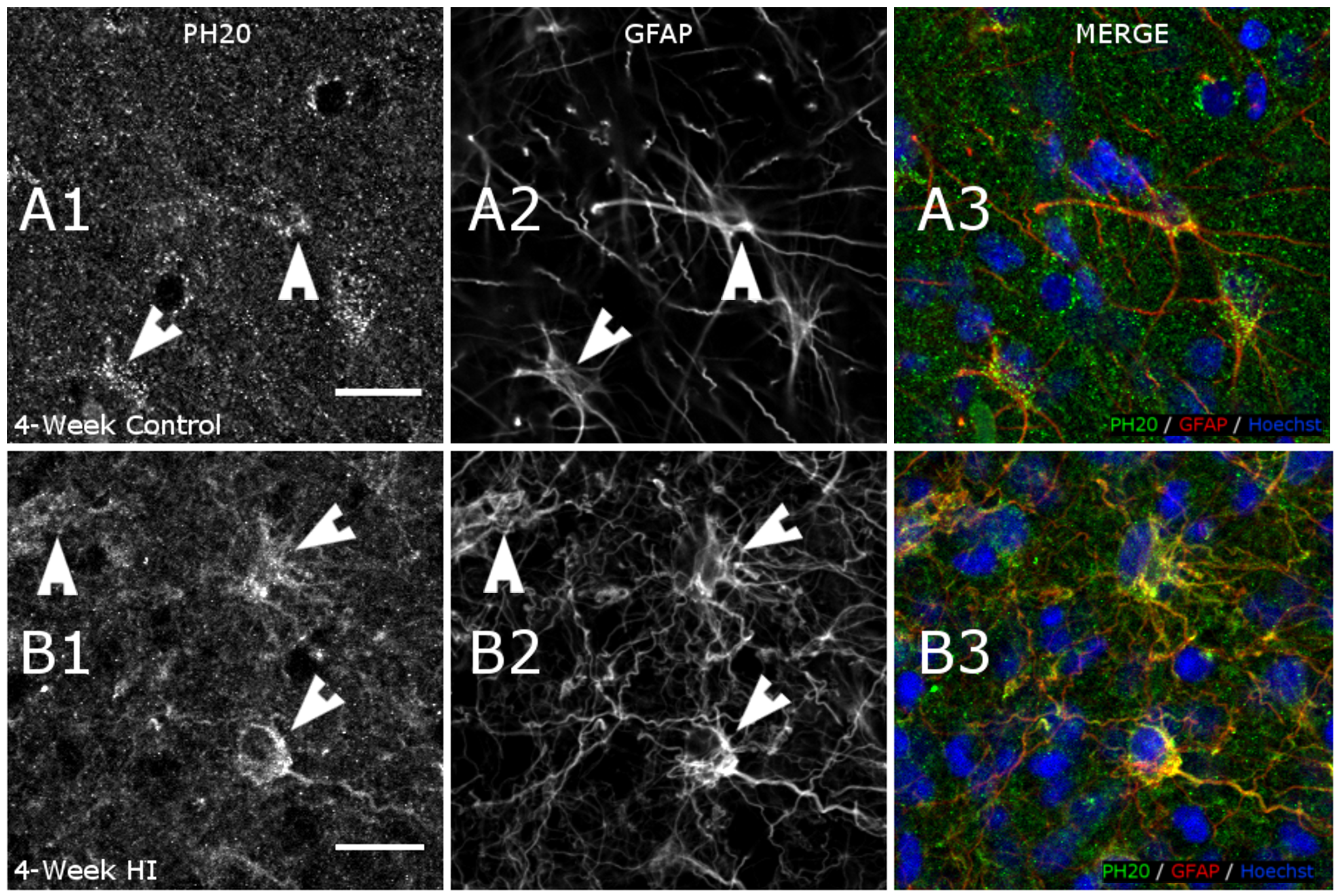

Supplement: Figure S2 — PH20 expression is persistently elevated in astrocytes four weeks after a single episode of HI at 93 dGa. (A) 4-week survival control: PH20 (A1), GFAP (A2), and merge (A3). (B) 4-week survival HI: PH20 (B1), GFAP (B2), and merge (B3). Mostly punctate PH20 staining co-localized (arrowheads) to GFAP-labeled astrocytes. Scale bars: 20 µm. (TIF) [file pone.0112800.s002.tif]

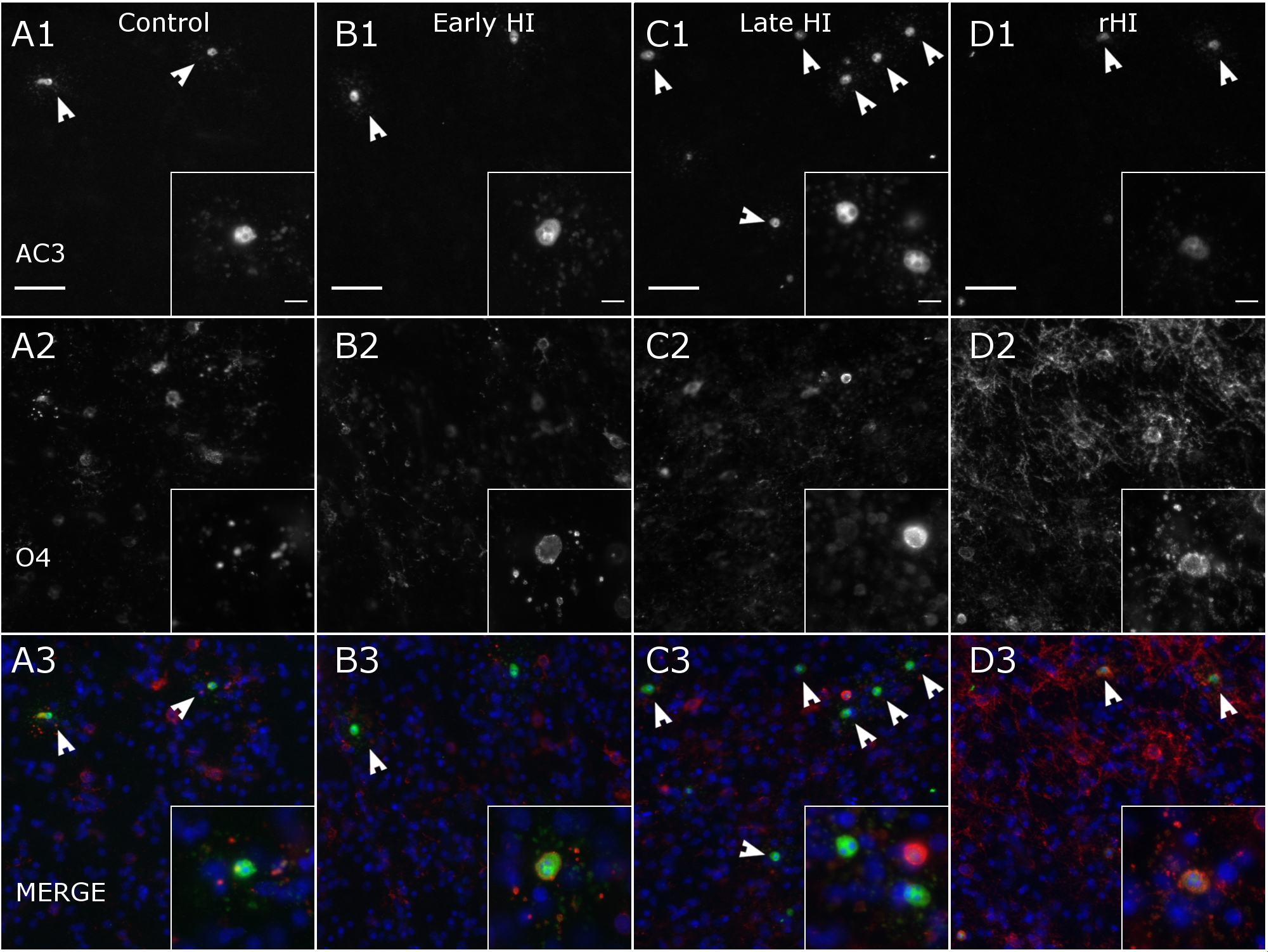

Supplement: Figure S3 — Typical appearance of activated caspase 3 (AC3; green) immunohistochemical staining in degenerating O4 antibody-labeled oligodendrocyte lineage cells (red) in control (A-panels), early HI (B-panels), late HI (C-panels) and rHI (D-panels). The images illustrate the range of appearances of degenerating cells. Nuclear morphology is visualized with Hoechst 33342 fluorescent counterstain (blue). Some cells displayed a halo of few degenerating processes that were very fragmented (e.g., panels A3 and B3). Other cells displayed a complete loss of processes and were shrunken in appearance with condensed fragmented chromatin (e.g., panel C3). Cells at early stages of degeneration displayed numerous fragmented processes (e.g., panel D3) and nuclear morphology notable for multiple balls of condensed chromatin, as also supported by the appearance of the AC3 staining in D1. (TIF) [file pone.0112800.s003.tif]
