# Supplementary material for: Role of Recurrent Hypoxia-Ischemia in Preterm White Matter Injury Severity
Source: PLoS One. 2014 Nov 12;9(11):e112800. doi: 10.1371/journal.pone.0112800 (PMC4229227; doi:10.1371/journal.pone.0112800)
Supplement: Table S1 — Summary of Sheep Physiological Responses. Physiological response to HI or rHI. Mean ± standard deviation. *p<0.05 within group vs. baseline (ANOVA or ANOVA on ranks if variances were unequal. Post hoc testing was Tukey as appropriate). ‡p<0.05 for a measure between groups (first and second HI were analyzed separately, using ANOVA/ANOVA on ranks with post hoc Tukey tests, and Student’s t-test respectively). **Indicates data could not be measured. †Late HI fetuses were not rendered ischemic during the first HI procedure, explaining many of our differences between groups. Note that because control fetuses were not instrumented, data are not available for them; however, late HI fetuses were only exposed to hypoxemia during the first insult, which allowed the effect of hypoxemia to be assessed in isolation. (PDF) [file pone.0112800.s004.pdf]

Blood Oxygen (CtO_2 Vol %)

		Baseline	Hypoxia	Hypoxia-Ischemia	Recovery
	Early HI	8.32 ± 0.73	5.35 ± 1.26*	7.86 ± 1.99[‡]	9.30 ± 0.91
	Late HI[†]	7.93 ± 1.13	3.78 ± 1.71*	4.18 ± 1.14*[‡]	7.98 ± 1.57
First HI	rHI	7.27 ± 0.79	2.86 ± 1.06*	5.20 ± 2.35	8.12 ± 0.91
	Late HI	7.50 ± 0.90	4.25 ± 1.61	5.35 ± 2.54	8.78 ± 1.66
Second HI	rHI	7.68 ± 1.48	4.42 ± 1.86	6.04 ± 2.87	8.48 ± 1.80

Oxygen Saturation (%)

		Baseline	Hypoxia	Hypoxia-Ischemia	Recovery
	Early HI	68.54 ± 4.72	45.10 ± 10.41*	57.00 ± 11.17[‡]	75.40 ± 2.94
	Late HI[†]	67.52 ± 9.35	31.83 ± 14.47*	32.92 ± 11.29*[‡]	67.47 ± 15.33
First HI	rHI	66.02 ± 12.79	25.52 ± 12.62*	37.32 ± 14.10*	70.50 ± 13.70
	Late HI	65.28 ± 9.66	36.65 ± 13.95	40.98 ± 20.47	67.08 ± 10.06
Second HI	rHI	60.16 ± 15.41	36.40 ± 19.96	45.42 ± 26.57	63.46 ± 16.31

Hemoglobin (g / dL)

		Baseline	Hypoxia	Hypoxia-Ischemia	Recovery
	Early HI	9.02 ± 0.76	8.73 ± 0.51	10.06 ± 1.05	9.40 ± 0.82
	Late HI[†]	8.80 ± 1.57	8.92 ± 1.57	9.66 ± 1.07	8.93 ± 1.61
First HI	rHI	8.42 ± 1.77	8.76 ± 1.99	9.82 ± 1.96	8.85 ± 1.68
	Late HI	8.65 ± 1.72	8.75 ± 1.81	10.05 ± 2.08	9.98 ± 2.48
Second HI	rHI	10.20 ± 4.10	10.50 ± 4.49	11.52 ± 4.64	10.66 ± 4.34

CO_2 pressure (mmHg)

		Baseline	Hypoxia	Hypoxia-Ischemia	Recovery
	Early HI	48.24 ± 3.50	43.68 ± 3.73	46.42 ± 2.22	48.55 ± 1.20
	Late HI[†]	48.38 ± 1.36	46.03 ± 4.34	45.26 ± 7.04	46.97 ± 2.43
First HI	rHI	49.47 ± 1.93	45.72 ± 3.98	48.06 ± 3.22	48.80 ± 2.14
	Late HI	48.08 ± 2.80	47.18 ± 0.94	47.33 ± 0.96	50.44 ± 2.32
Second HI	rHI	49.24 ± 3.63	48.58 ± 3.23	48.82 ± 2.02	50.22 ± 2.84

Oxygen Pressure (mmHg)

		Baseline	Hypoxia	Hypoxia-Ischemia	Recovery
	Early HI	25.90 ± 2.49	18.33 ± 2.63*	22.78 ± 4.31[‡]	29.05 ± 0.79
	Late HI[†]	25.43 ± 3.85	14.85 ± 3.66*	15.00 ± 2.71*[‡]	26.38 ± 5.73
First HI	rHI	24.93 ± 4.76	13.04 ± 3.30	17.20 ± 3.44	28.48 ± 5.24
	Late HI	24.88 ± 3.73	16.90 ± 3.83	18.30 ± 5.00	28.64 ± 3.92
Second HI	rHI	25.74 ± 5.57	23.06 ± 14.57	20.42 ± 5.97	28.52 ± 4.07

Hematocrit (%)

		Baseline	Hypoxia	Hypoxia-Ischemia	Recovery
	Early HI	27.96 ± 2.23	27.15 ± 1.50	31.12 ± 3.16	29.05 ± 2.51
	Late HI[†]	27.35 ± 4.75	27.63 ± 4.64	29.88 ± 3.13	27.77 ± 4.80
First HI	rHI	26.18 ± 5.41	27.22 ± 6.07	30.42 ± 5.89	27.47 ± 5.01
	Late HI	26.85 ± 5.25	26.93 ± 6.71	31.85 ± 6.73	30.82 ± 7.46
Second HI	rHI	31.58 ± 12.39	32.52 ± 13.67	35.48 ± 13.99	32.98 ± 13.13

		Baseline	Hypoxia	Hypoxia-Ischemia	Recovery
**Blood Glucose (mmol / L)**					
First HI	Early HI	1.36 ± 0.21	1.45 ± 0.21	1.76 ± 0.27	1.53 ± 0.15
	Late HI†	1.27 ± 0.32	1.30 ± 0.28	1.38 ± 0.34	1.52 ± 0.38
	rHI	1.28 ± 0.31	1.08 ± 0.30	1.64 ± 0.36	1.50 ± 0.46
Second HI	Late HI	1.20 ± 0.16	1.23 ± 0.21	1.73 ± 0.21*	1.58 ± 0.26
	rHI	1.14 ± 0.25	1.16 ± 0.27*	1.70 ± 0.34	1.62 ± 0.28
**Blood Lactate (mmol / L)**					
First HI	Early HI	1.10 ± 0.19	1.20 ± 0.24	2.56 ± 0.57*‡	3.83 ± 0.59*
	Late HI†	1.35 ± 0.34	1.52 ± 0.44	2.84 ± 1.06*‡	3.08 ± 1.32*‡
	rHI	1.32 ± 0.38	1.90 ± 0.85	4.59 ± 0.74*‡	4.78 ± 0.87*‡
Second HI	Late HI	1.33 ± 0.24	1.43 ± 0.13	4.20 ± 1.35	4.90 ± 1.75
	rHI	1.34 ± 0.51	1.36 ± 0.38	3.88 ± 1.62	5.02 ± 2.07
**pH**					
First HI	Early HI	7.38 ± 0.02	7.41 ± 0.03	7.36 ± 0.03	7.34 ± 0.03
	Late HI†	7.38 ± 0.01	7.41 ± 0.03	7.37 ± 0.04	7.36 ± 0.03
	rHI	7.39 ± 0.01	7.41 ± 0.04	7.35 ± 0.04	7.32 ± 0.03*
Second HI	Late HI	7.37 ± 0.03	7.38 ± 0.02	7.32 ± 0.05	7.29 ± 0.06
	rHI	7.37 ± 0.03	7.38 ± 0.03	7.33 ± 0.05	7.29 ± 0.07
**Heart Rate (BPM)**					
First HI	Early HI	196.86 ± 9.33	201.54 ± 12.57	**	251.02 ± 60.93
	Late HI†	207.00 ± 11.49	238.50 ± 25.16	**	224.00 ± 19.29
	rHI	214.50 ± 51.00	213.00 ± 35.83	**	229.50 ± 21.56
Second HI	Late HI	200.00 ± 9.17	196.00 ± 9.17	**	226.00 ± 12.49
	rHI	196.50 ± 13.30	217.50 ± 39.31	**	226.50 ± 16.52
**Blood Pressure (mmHg)**					
First HI	Early HI	33.13 ± 3.64	33.13 ± 3.64	**	31.75 ± 3.69
	Late HI†	35.24 ± 6.08	35.24 ± 6.08	32.23 ± 4.52‡	34.50 ± 1.40
	rHI	34.99 ± 6.18	34.99 ± 6.18	5.04 ± 1.22*‡	33.93 ± 5.09
Second HI	Late HI	28.94 ± 2.61	28.94 ± 2.61	6.11 ± 3.31*	30.86 ± 2.22
	rHI	33.20 ± 5.05	33.20 ± 5.05	5.53 ± 1.37*	33.16 ± 2.40
